# Exploring the feasibility of using mice as a substitute model for investigating microglia in aging and Alzheimer’s disease though single cell analysis

**DOI:** 10.1371/journal.pone.0311374

**Published:** 2024-11-26

**Authors:** Rong He, Qiang Zhang, Limei Wang, Yiwen Hu, Yue Qiu, Jia Liu, Dingyun You, Jishuai Cheng, Xue Cao

**Affiliations:** 1 Laboratory Animal Department, Kunming Medical University, Kunming, Yunnan, China; 2 Dermatology Department of Xiangya Hospital, Central South University, Changsha, Hunan, China; 3 School of Public Health, Kunming Medical University, Kunming, Yunnan, China; Huashan Hospital Fudan University, CHINA

## Abstract

**Objective:**

To guide animal experiments, we investigated the similarities and differences between humans and mice in aging and Alzheimer’s disease (AD) at the single-nucleus RNA sequencing (snRNA-seq) or single-cell RNA sequencing (scRNA-seq) level.

**Methods:**

Microglia cells were extracted from dataset GSE198323 of human post-mortem hippocampus. The distributions and proportions of microglia subpopulation cell numbers related to AD or age were compared. This comparison was done between GSE198323 for humans and GSE127892 for mice, respectively. The Seurat R package and harmony R package were used for data analysis and batch effect correction. Differentially expressed genes (DEGs) were identified by FindMarkers function with MAST test. Comparative analyses were conducted on shared genes in DEGs associated with age and AD. The analyses were done between human and mouse using various bioinformatics techniques. The analysis of genes in DEGs related to age was conducted. Similarly, the analysis of genes in DEGs related to AD was performed. Cross-species analyses were conducted using orthologous genes. Comparative analyses of pseudotime between humans and mice were performed using Monocle2.

**Results:**

(1) **Similarities:** The proportion of microglial subpopulation Cell_APOE/Apoe shows consistent trends, whether in AD or normal control (NC) groups in both humans and mice. The proportion of Cell_CX3CR1/Cx3cr1, representing homeostatic microglia, remains stable with age in NC groups across species. Tuberculosis and Fc gamma R-mediated phagocytosis pathways are shared in microglia responses to age and AD across species, respectively. (2) **Differences:** IL1RAPL1 and SPP1 as marker genes are more identifiable in human microglia compared to their mouse counterparts. Most genes of DEGs associated with age or AD exhibit different trends between humans and mice. Pseudotime analyses demonstrate varying cell density trends in microglial subpopulations, depending on age or AD across species.

**Conclusions:**

Mouse Apoe and Cell_Apoe maybe serve as proxies for studying human AD, while Cx3cr1 and Cell_Cx3cr1 are suitable for human aging studies. However, AD mouse models (App^_NL_G_F^) have limitations in studying human genes like IL1RAPL1 and SPP1 related to AD. Thus, mouse models cannot fully replace human samples for AD and aging research.

## 1 Introduction

With the aging population in China, research on aging and diseases like dementia have gained significant attention. Due to genetic homologies, mice and transgenic mouse models are widely used to study human biology and diseases [1, 2]. In Alzheimer’s disease (AD) research, various mouse models have been developed. The first-generation models (APP/PS1) typically involve the overexpression of mutated forms of the amyloid precursor protein (APP) or APP/presenilin 1 (PS1) cDNA, which result in the accumulation of abnormal amyloid in the mouse brain through genetic engineering. These mice exhibit symptoms similar to AD. The second-generation models (APP^_NL_F^ and APP^_NL_G_F^), are knock-in mice that carry mutations, such as Swedish, Beyreuther/Iberian, and optionally Arctic, in the APP gene. These mutations closely resemble those found in human AD. The third-generation mouse models (APP^_NL_F^ and Psen1P117L/WT), incorporate mutations in the APP gene alongside the P117L mutation in the PS1 gene. PS1 plays a pivotal role in AD development, and the P117L mutation in the model corresponds to a common mutation associated with human AD. The third-generation mouse models more comprehensively simulate human AD genetic and environmental factors, complex pathologies, and gene mutation interactions. Therefore, these models replicate the pathogenesis and cognitive impairments of AD that are similar to those in humans [1, 3, 4]. Similarly, naturally aging mice, mirroring the cognitive decline seen in the elderly, are also employed in AD research. However, an increasing number of studies suggests that neither transgenic nor naturally aging mice models perfectly replicate all the changes observed in AD patients [5–7].

Microglia, the immune cells of the central nervous system [8], play multifaceted roles, significantly influencing brain development, maintenance, and injury repair, as well as defending against pathogens [9, 10]. They contribute to the formation of neuronal circuits by pruning synapses and removing apoptotic cells [10]. Regarding AD, microglia display a dual role, including protective and triggering mechanisms. On one hand, microglia can reduce amyloid-beta (Aβ) plaque toxicity through phagocytosis [8]. On the other hand, in AD patients, microglia display a chronic inflammatory response related to abnormal activation, which might exacerbate neuronal damage and contribute to the formation of neurofibrillary tangles, key features of AD [8, 11, 12]. Moreover, a significant majority of AD risk genes are highly expressed in microglia within the brain [8]. Understanding how microglia influence AD progression is essential for developing targeted therapies.

Transgenic mice and naturally aging mouse models always are employed to investigate the impact of microglia on AD. However, there are studies suggesting that there exist significant differences between mouse and human immune systems [2, 13], and researchers also hold that there are obvious differences between the microglial cells of mice and those of humans [10, 14]. These differences raise concerns about the applicability of mouse models for investigating the specific role of microglia in human AD. However, no current studies have thoroughly elucidated the precise similarities and differences in microglial cells between mouse AD models and human AD patients, particularly with respect to single-cell level findings.

This study bridges these gaps by systematically comparing microglia-related datasets at the single-cell level between humans and mice. It focuses on three aspects: (1) identifying similarities and differences in microglial subpopulations and genes across different age groups in normal states; (2) comparing these aspects between normal and transgenic mice, and between normal and AD-affected humans in older age; and (3) examining changes in microglial subpopulations and genes considering both age and AD. The results indicate that while mouse models provide valuable insights, they can not fully replace human samples in AD and aging research.

## 2 Materials and methods

**Fig 1:**
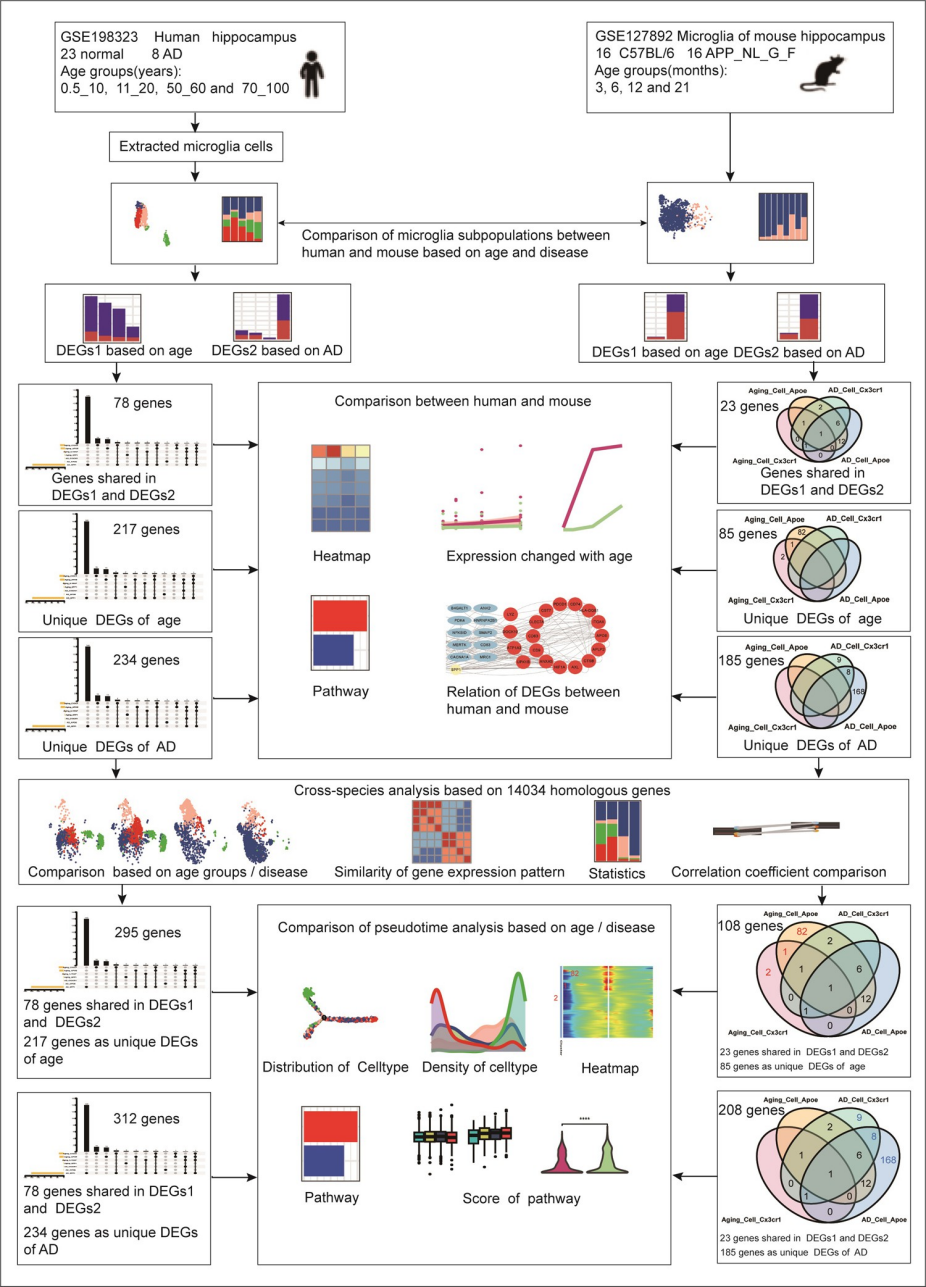
Data analysis workflow for microglia comparison in humans and mice.

A comprehensive analytical workflow was developed to systematically compare microglia between humans and mice (Fig 1), encompassing five key steps: (1) Microglial cells were obtained from two datasets: GSE198323 [15], consisting of 31 human post-mortem hippocampal specimens, and GSE127892 [16], comprising 16 mouse hippocampus samples and isolated microglia. (2) Subpopulations of microglia were compared based on age and AD status in humans and mice. (3) Shared DEGs related to age and AD across humans and mice were examined, along with genes associated with age but not AD, and those associated with AD but not age, in both species.(4) Cross-Species analysis using homologous genes were analyzed to identify cross-species similarities and differences. (5) Comparative pseudotime analyses were conducted based on age and AD status. The materials and methods used for data analysis were detailed in the supplementary materials.

## 3 Results

### 3.1 Data acquisition, quality control, cell type identification and microglia extraction

Data Acquisition. We downloaded a snRNA-seq dataset (GSE198323) of the human hippocampus from the Gene Expression Omnibus (GEO) database, including 23 normal control (NC) and 8 AD samples, with ages ranging from 0.6 to 92 years (S1 Table). Another scRNA-seq dataset (GSE127892) of microglial cells from mouse hippocampus was also downloaded, comprising 8 C57BL/6 wild-type and 8 APP^_NL_G_F^ mice at four time points: 3, 6, 12, and 21 months.

Quality Control. Quality control (QC) procedures were executed on both the human GSE198323 and mouse GSE127892 datasets (S1A-S1L Fig in S1 Appendix). After QC, the GSE198323 contained 124,119 cells and 32,614 genes (AD: 34,080 cells, NC: 90,039 cells). The GSE127892 included 5,432 cells with 29,025 genes in microglial cells (APP^_NL_F_G^: 2,669 cells, C57BL/6: 2,763 cells).

Cell Type Identification and Microglia Extraction. Analysis of cell types within the human hippocampus dataset revealed nine distinct cell types, along with marker genes identified from previous studies [15–18] (S2A, S2B Fig in S1 Appendix). These included astrocytes (Astro, 15,674 cells, AQP4), endothelial cells (Endo, 1,211 cells, FLT1), microglia (Micro, 5,552 cells, APBB1IP), oligodendrocytes (Oligo, 30,841 cells, MOBP), oligodendrocyte progenitor cells (OPCs, 9,713 cells, VCAN), dentate gyrus neurons (Gran, 13,035 cells, PROX1), pyramidal neurons (Pyram, 2,018 cells, TSHZ2), inhibitory neurons (In, 10,445 cells, GAD2), and a subtype of excitatory neurons (ExN.sub, 33,013 cells). Neuronal markers such as SYT1 for neurons and NEUROD2 for excitatory neurons were observed (S2C Fig in S1 Appendix), which facilitated the classification of excitatory neurons, excluding Gran and Pyram, as ExN.sub. Human microglia were isolated for subsequent analysis and comparison with mouse microglia. Notably, no isolation was required for the mouse dataset, as it exclusively contained microglial cells.

### 3.2 Comparative analysis of microglia subpopulations between humans and mice

In the human dataset (GSE198323), four microglia subpopulations were identified using marker genes, and their characteristics were described. These subpopulations were distinguished by marker genes: CX3CR1 and APOE for homeostatic and disease-associated microglia (DAM), respectively [10], and IL1RAPL1 and SPP1 for additional subpopulations. The identified subpopulations included Cell_CX3CR1 (36.5%, 2026 cells), Cell_APOE (13.5%, 751 cells), Cell_IL1RAPL1 (23.1%, 1285 cells), and Cell_SPP1 (26.9%, 1490 cells), as visualized with Uniform Manifold Approximation and Projection (UMAP) plots (S3A, S3B Fig in S1 Appendix). Each subpopulation exhibited distinct marker gene expressions, such as heightened CX3CR1 in Cell_CX3CR1 and elevated APOE in Cell_APOE [10]. Cell_IL1RAPL1 and Cell_SPP1 were heightened expression of IL1RAPL1 and SPP1, respectively (S3C Fig in S1 Appendix) [10].

In the mouse dataset GSE127892, two distinct subpopulations, namely Cell_Cx3cr1 and Cell_Apoe were identified based on their marker genes. In this dataset, two subpopulations were found: Cell_Cx3cr1 (76.3%, 4145 cells) and Cell_Apoe (23.7%, 1287 cells). These subpopulations were visualized using UMAP plots (S3D, S3E Fig in S1 Appendix), with Cell_Cx3cr1 and Cell_Apoe showing high expressions of Cx3cr1 and Apoe, respectively (S3F Fig in S1 Appendix). While the expression of Il1rapl1 was limited, the expression of Spp1 was slightly higher in Cell_Apoe, indicating that Cell_IL1RAPL1 and Cell_SPP1 might be more easily identified in humans than those in mice.

**Fig 2:**
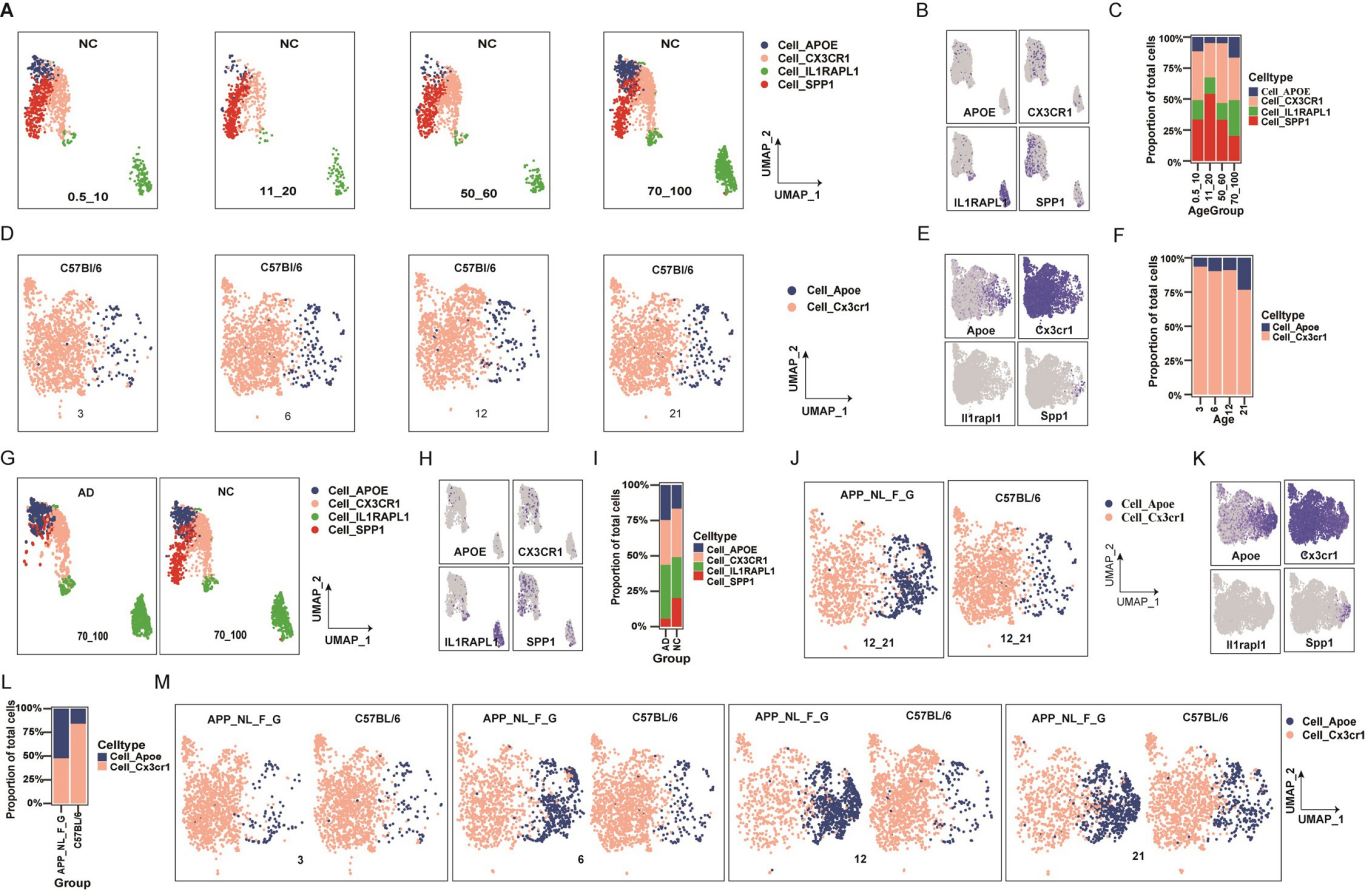
Comparative analysis of microglial subpopulations in humans and mice. A. Age-group-based distribution of four major microglial subpopulations in human normal samples (GSE198323). B. Marker gene (CX3CR1, APOE, IL1RAPL1, SPP1) distributions for these subpopulations in human normal groups. C. Age-group-based comparison of microglial subpopulation proportions in human normal samples. D. Age-group-based distribution of two major microglial subpopulations in mouse normal samples (GSE127892). E. Marker gene (Cx3cr1, Apoe) distributions for these subpopulations, and Il1rapl1 and Spp1 distributions in mouse normal samples. F. Age-group-based comparison of microglial subpopulation proportions in mouse normal samples. G. Disease-status-based distribution of four major microglial subpopulations in human samples aged 70–100 (GSE198323). H. Marker gene distributions for human microglial subpopulations in normal and AD groups aged 70–100. I. Disease-status-based comparison of microglial subpopulation proportions in the 70–100 age group. J. Disease-status-based distribution of two major microglial subpopulations in mice at 12 and 21 months (GSE127892). K. Marker gene distributions for mouse microglial subpopulations, and Il1rapl1 and Spp1 distributions in mice at 12 and 21 months. L. Disease-status-based comparison of microglial subpopulation proportions at 12 and 21 months. M. Age- and disease status-based distributions of two microglial subpopulations in mice.

Comparisons of microglial subpopulations were performed across different age groups. Microglial subpopulation distribution and marker gene distribution, along with the proportion of cell numbers in human and mouse normal groups, were visualized by using UMAP plots (Fig 2A–2F). Cell_CX3CR1/Cx3cr1 and Cell_APOE/Apoe were consistently identified by their respective marker genes across age groups (S3C-S3F Fig in S1 Appendix). The distribution of Cell_CX3CR1/Cx3cr1 remained stable across age groups in normal conditions for both species, with the exception of humans aged 11–20 and mice aged 21 months (Fig 2A, 2C, 2D and 2F). The distribution of Cell_APOE/Apoe increased with age in both species (Fig 2A and 2D).

Disease Status-related comparison of microglial subpopulations. Distributions of microglial subpopulations and maker gene were visualized based on disease status in both humans and mice, respectively (Fig 2G, 2H, 2J and 2K), with cell number percentages calculated (Fig 2I and 2L). Different trends were observed for Cell_CX3CR1/Cx3cr1 in AD vs. NC groups: proportions remained similar in humans aged 70–100 but were lower in mice AD groups (Fig 2I and 2K). Cell_APOE/Apoe distributions showed higher proportions in AD groups compared to NC in both species. In humans, Cell_IL1RAPL1 had higher proportions in AD groups aged 70–100 (Fig 2I), while Cell_SPP1 had lower distributions in AD groups compared to NC aged 70–100 (Fig 2G and 2I).

Age- and disease status-related comparison of microglial subpopulations in mice. The analysis of microglial subpopulations between APP^_NL_F_G^ and C57BL/6 mice across different age groups revealed distinct patterns influenced by age and disease status (Fig 2M). In C57BL/6 mice, the distribution of Cell_Apoe increased with age, a trend also observed in APP^_NL_F_G^ mice. This suggested an interaction between age and AD, implying that both factors might contribute to AD progression. Age was a pivotal risk factor for AD, affecting both the general and hereditary AD populations [10, 19]. Consequently, our subsequent research focused on factors that change in conjunction with age and AD.

### 3.3 DEGs associated with age and AD in humans

**Fig 3:**
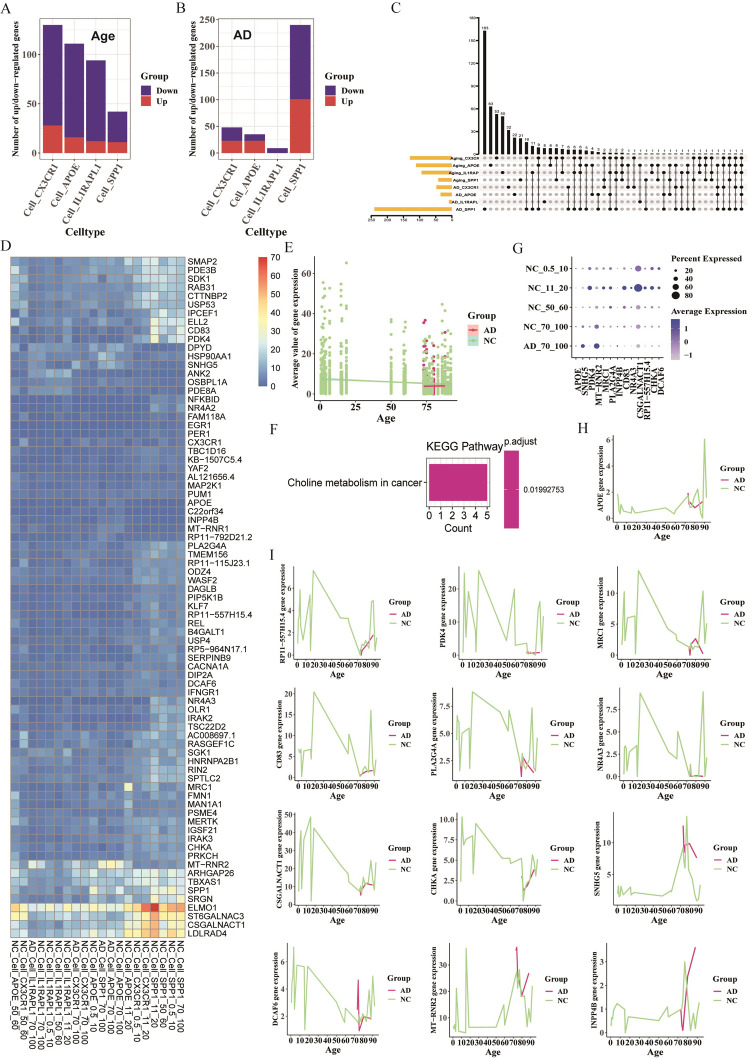
Shared DEGs associated with age and AD in humans (GSE198323). A. Number of up-regulated and down-regulated DEGs between younger (ages 0.5–10, 11–20) and older (ages 70–100) groups for microglia subpopulations. B. Number of up-regulated and down-regulated DEGs between AD and NC groups in the 70–100 age group for microglia subpopulations. C. Upset plot showing shared genes for age- and AD-related DEGs. D. Heatmap displaying average expressions of 78 shared genes from age-related DEGs (DEGs1) and disease-related DEGs (DEGs2) (p-value < 0.05 and |avg_log2FC| > 0.5). E. Scatterplot with linearly fitted curve showing average expression of 78 shared genes from DEGs1 and DEGs2 varying with age. F. KEGG enrichment analysis using the 78 shared genes from DEGs1 and DEGs2. G. Dot plot exhibiting 13 genes (12 shared genes from DEGs1 and DEGs2, plus APOE) (p-value < 0.05 and |avg_log2FC| > 1). H. Line chart showing APOE expression variation with age in both NC and AD groups. I. Line charts displaying expression variations of 12 shared genes from DEGs1 and DEGs2 with age in both NC and AD groups.

To explore the characteristics of shared genes for DEGs related to age and AD in humans, a detailed analysis was conducted as follows:

Acquisition and Comparison of DEGs. DEGs linked to age were identified for the four microglia subpopulations, with more down-regulated than up-regulated DEGs (Fig 3A). DEGs associated with AD were also identified, showing fewer AD-related DEGs compared to age-related DEGs, except for Cell_SPP1 (Fig 3B).

Screening and Characterizing DEGs. A total of 78 shared DEGs (16+11+9+6×2+5+4+2×3+1×15 = 78) related to both age and AD were screened using filter criteria (p-value < 0.05 and |avg_log2FC| > 0.5), visualized with an upset graph (Fig 3C and S2 Table). These genes were displayed in a heatmap (Fig 3D), showing a significant negative correlation with age in the NC group (R = -0.144, P < 0.05) but a non-significant positive correlation in the AD group (R = 0.02, P = 0.72) (Fig 3E). KEGG enrichment analysis highlighted the Choline metabolism in cancer pathway (Fig 3F).

Visualization of DEGs. 12 shared DEGs (4+2×3+1×2 = 12) were screened with criteria (p-value < 0.05 and |avg_log2FC| > 1) and visualized (Fig 3G). Despite APOE was not one of these DEGs, it was included due to its role as a marker gene for DAM-like microglia in AD (Fig 3G). The expression changes of these 13 genes in the AD and NC groups across different ages were represented using line charts (Fig 3H and 3I). In the NC group, genes like APOE, RP11-557H15.4, PDK4, MRC1, CD83, PLA2G4A, and NR4A3 showed more significant age-related expression changes. In the AD group, the expression of CSGALNACT1, CHKA and SNHG5 remained stable, while DCAF6, MT-RNR2, and INPP4B showed more significant changes with age within the 70–100 age range.

### 3.4 DEGs related to age and AD in mice

**Fig 4:**
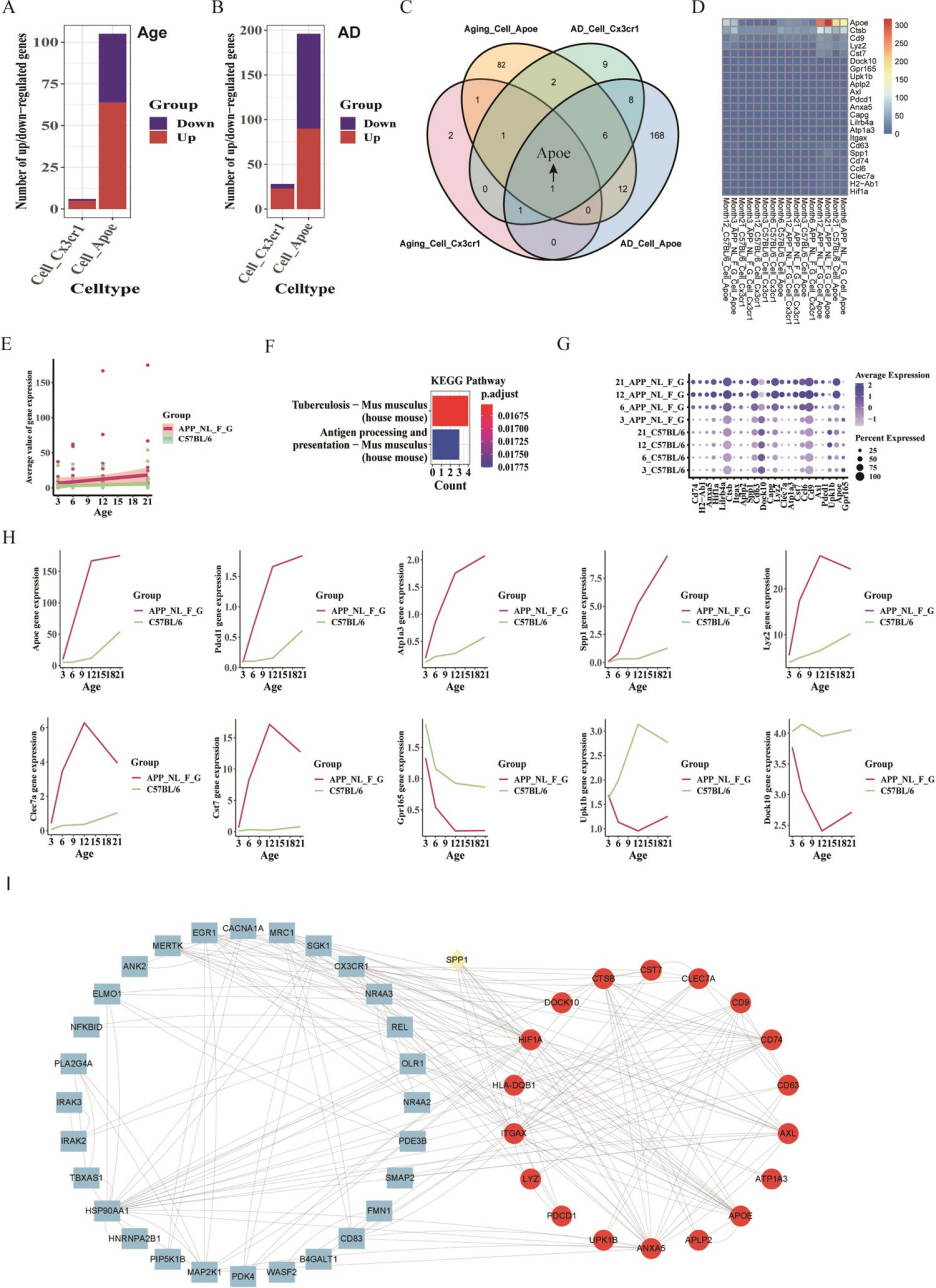
Shared DEGs in mice related to age and AD (GSE127892). A. Numbers of up-regulated and down-regulated DEGs in microglia subpopulations between younger (3, 6 months) and older (21 months) groups. B. Numbers of up-regulated and down-regulated DEGs in microglia subpopulations between the APP^_NL_F_G^ and C57BL/6 groups at 12 and 21 months. C. Venn diagram for screening shared genes from age- or AD-related DEGs. D. Heatmap showing average expressions of 23 shared genes from age-related DEGs (DEGs1) and disease-related DEGs (DEGs2) (p-value < 0.05 and |avg_log2FC| > 0.5). E. Scatterplot with a linearly fitted curve showing the average expression of 23 shared genes from DEGs1 and DEGs2 varying with age. F. KEGG enrichment analyses using 23 shared genes from DEGs1 and DEGs2. G. Dot plot exhibiting 23 shared genes from DEGs1 and DEGs2 (p-value < 0.05 and |avg_log2FC| > 0.5). H. Line charts showing the average expressions of 10 shared genes from DEGs1 and DEGs2 varying with age in C57BL/6 and APP^_NL_F_G^ groups. I. 28 DEGs in humans (blue elliptical shape), 18 genes in mice (red circle shape), and 1 gene (SPP1, yellow circle shape) shared between humans and mice, illustrating the close relationship between species.

To investigate the characteristics of shared genes for DEGs associated with age and AD in mice, we further performed a comprehensive analysis as follows:

Acquisition and Comparison of DEGs. DEGs related to age were detected for two microglia subpopulations (Fig 4A). DEGs associated with AD were also identified for the same subpopulations (Fig 4B). Notably, the number of AD-related DEGs in Cell_Apoe exceeded those related to age, while age-related DEGs in Cell_Cx3cr1 were fewer compared to AD-related DEGs.

Screening and Characterizing DEGs. A Venn diagram was used to identify shared genes among DEGs related to age and AD (p-value < 0.05 and |avg_log2FC| > 0.5), with Apoe being a common gene in both Cell_Cx3cr1 and Cell_Apoe (Fig 4C). A heatmap depicted the expression of 23 shared genes (12+1+1+1+6+2 = 23, Fig 4D). Apoe expression was higher in Cell_Apoe of the APP^_NL_F_G^ group at 6, 12, and 21 months and in the C57BL/6 group at 21 months. The average expressions of these genes showed a positive correlation with age in both NC and AD groups, though this was not statistically significant (NC: R = 0.135, P = 0.198; AD: R = 0.161, P = 0.126) (Fig 4E). KEGG enrichment analyses highlighted pathways, including tuberculosis and antigen processing and presentation (Fig 4F).

Visualization of DEGs. The expressions of the 23 shared genes were displayed by a dot plot, grouped by age and AD (Fig 4G). Line charts showed variations in gene expressions with age in both AD and NC groups (Fig 4H and S4A Fig in S1 Appendix). At 3 months, the expressions were similar between APP^_NL_F_G^ and C57BL/6 mice, but changed significantly in APP^_NL_F_G^ mice at 6 months. Genes like Gpr165, Upk1b, and Dock10 had higher expressions in the C57BL/6 group compared to APP^_NL_F_G^. Most of the 23 shared genes showed lower expressions in the NC group compared to the AD group with age advancement. These results indicated that the expression of most age- and disease-related DEGs was higher in the APP^_NL_F_G^ group than in the C57BL/6 group with age.

### 3.5 Similarities and differences in DEGs relevant to age or AD in microglial cells between humans and mice

DEGs related to age and AD exhibited similar traits in humans and mice. (1) In both humans and mice, the average expressions of genes in DEGs shared between age and AD groups showed a positive correlation with age within the AD groups, although these correlations were not statistically significant (P>0.05). (Figs 3E and 4E). (2) There was a notable association between human and mouse DEGs related to age and AD. Specifically, 28 human genes (blue ellipse), 18 mouse genes (red circle), and 1 shared gene (SPP1, yellow circle) exhibited strong cross-species relevance (Fig 4I).

Number of gene in DEGs related to age and AD exhibited different traits in humans and mice. (1) Humans had more age-related DEGs in the CELL_CX3CR1 subpopulation compared to mice’s CELL_Cx3cr1. In humans, down-regulated genes in age-related DEGs for CELL_APOE outnumbered up-regulated genes, while mice displayed the opposite pattern (Figs 3A and 4A and S5A and S5B Fig in S1 Appendix). For CELL_CX3CR1, humans showed an equal number of up- and down-regulated genes, whereas mice had more up-regulated genes. For CELL_APOE, humans had more up-regulated genes, while mice had more down-regulated genes (S5C, S5D Fig in S1 Appendix).

Gene expression trends in DEGs varied by age differently between humans and mice. (1) In the AD group, human APOE expression showed less variation with age and was not part of age-related DEGs. In contrast, mouse Apoe expression showed more age-related variation and was included in DEGs (Figs 3G, 3H, 4D and 4H). (2) In humans, the average expression of shared genes in age- and AD-related DEGs negatively correlated with age, and was statistically significant (P<0.05, Fig 3E). Conversely, in C57BL/6 mice, this correlation was positive but not statistically significant (P>0.05, Fig 4E). (3) In humans, the expression of most genes in DEGs involved in age and AD was lower in the AD group than in the NC group (Fig 3H and 3I). In mice, the expression of these genes was higher in the AD group compared to the NC group (Fig 4H, S4A Fig in S1 Appendix).

### 3.6 Cross-species analyses of humans and mice

Cross-species analyses involving humans and mice were conducted. Integrated microglial cells from both species were analyzed using UMAP plots, revealing four subpopulations: Cell_CX3CR1 (59.6%), Cell_APOE (15.6%), Cell_IL1RAPL1 (9.9%), and Cell_SPP1 (14.9%) (S6A, S6D Fig in S1 Appendix). Classic marker genes were displayed in a dot plot (S6B, S6C Fig in S1 Appendix). UMAP plots showed species-specific distributions, with Cell_IL1RAPL1 and Cell_SPP1 being less prominent in mice, likely due to lower expression of Il1rapl1 and Spp1 in mouse microglia (S3C, S3F Fig in S1 Appendix). Consequently, these subpopulations were not identified in mice (S3D Fig in S1 Appendix). Despite differences in gene counts (44,344 in humans and 29,025 in mice), 14,034 homologous genes were identified using the homologene package in R. We speculated that when the number of homologous genes was fewer than that of mouse genes, Cell_IL1RAPL1 and Cell_SPP1 could be identified in mice using homologous marker genes.

**Fig 5:**
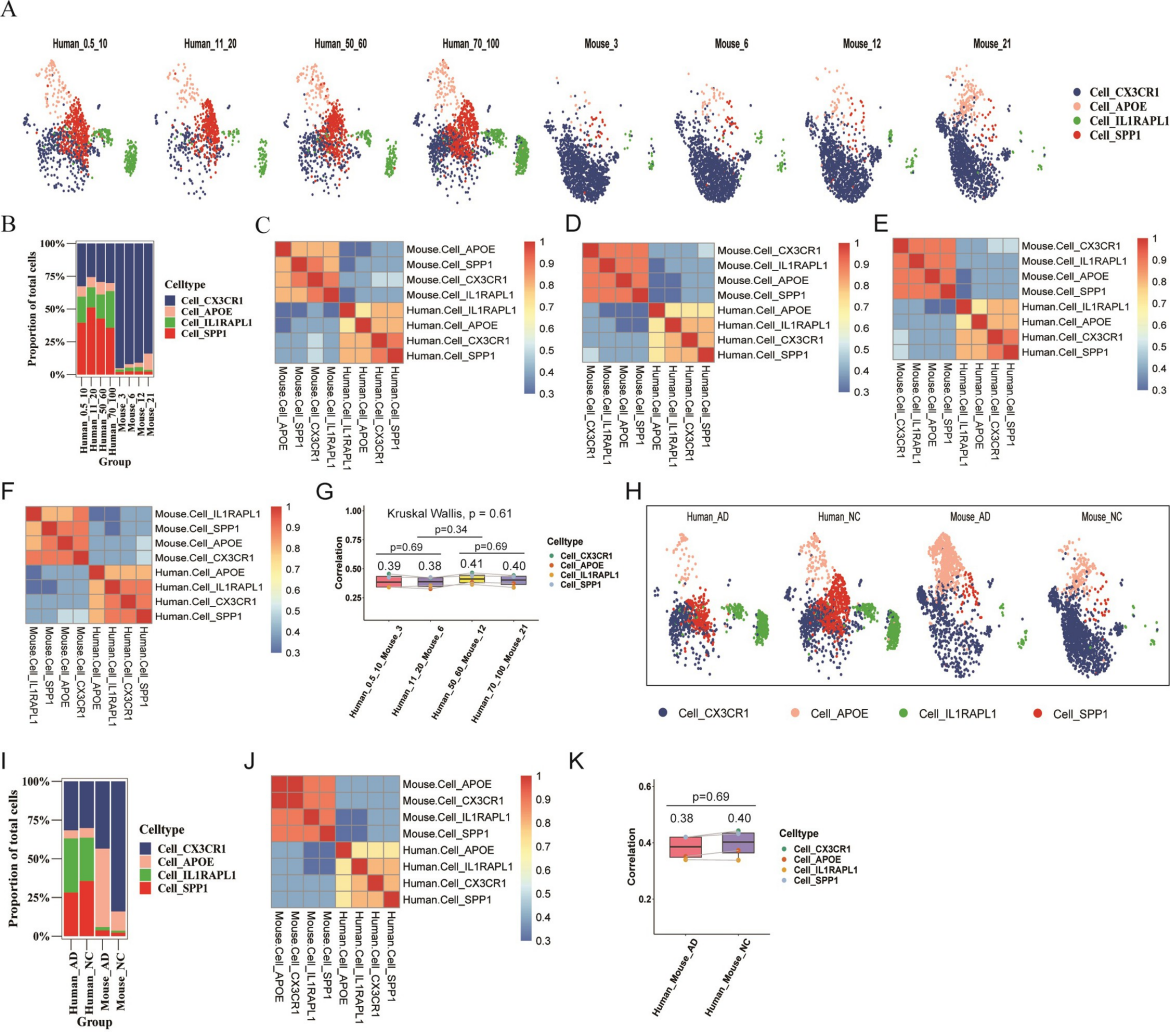
Cross-species analyses of humans and mice. A. UMAP plot displaying four major microglial subpopulations grouped by species and age groups. B. Proportions of cell numbers in the four subpopulations divided by species and age groups. C. Heatmap of correlation coefficients for gene expression in four microglial subpopulations between 0.5-10-year-old humans and 3-month-old mice. D. Heatmap of correlation coefficients for gene expression in four microglial subpopulations between 11-20-year-old humans and 6-month-old mice. E. Heatmap of correlation coefficients for gene expression in four microglial subpopulations between 50-60-year-old humans and 12-month-old mice. F. Heatmap of correlation coefficients for gene expression in four microglial subpopulations between 70-100-year-old humans and 21-month-old mice. G. Comparison of average correlation coefficients for four microglial subpopulations across four age groups between humans and mice. H. UMAP plot displaying four major microglial subpopulations grouped by species and disease status. I. Proportions of cell numbers in four subpopulations divided by species and disease status. J. Heatmap of correlation coefficients for gene expression in four microglial subpopulations between 70-100-year-old humans and 21-month-old AD mice. K. Comparison of average correlation coefficients for four microglial subpopulations between the AD and NC groups in humans and mice.

Age-Related Cross-Species Analyses in the NC Group. (1) UMAP plots showed species and age group-specific distributions, with humans having more Cell_APOE, Cell_IL1RAPL1, and Cell_SPP1, and mice having more Cell_CX3CR1 (Fig 5A). (2) Humans maintained steady proportions of Cell_CX3CR1 and Cell_APOE across age groups, while mice showed decreasing Cell_CX3CR1 and increasing Cell_APOE proportions with age. Cell_IL1RAPL1 and Cell_SPP1 proportions were consistently lower in mice (Fig 5B). (3) Gene expression patterns varied across age intervals. The average correlation coefficients for microglial subpopulations between humans and mice were 0.39, 0.38, 0.41, and 0.4 for corresponding age groups (Fig 5G. 0.5–10 years old humans vs. 3-month-old mice, Fig 5C, 11–20 years vs. 6-month-old, Fig 5D, 50–60 years vs. 12-month-old, Fig 5E, and 70–100 years vs. 21-month-old mice, Fig 5F, respectively), with no significant differences (Kruskal-Wallis test, p = 0.61; Mann-Whitney-Wilcoxon test, p = 0.69, 0.34, 0.69).

Cross-Species Analyses in NC and AD Groups (humans: 70–100 years old, mice: 21 months old).(1) UMAP plots indicated differing distribution trends of Cell_APOE between species and disease states. In AD mice, Cell_APOE increased compared to NC mice, while it decreased in AD humans compared to NC humans. Cell_IL1RAPL1 and Cell_SPP1 distributions were higher in humans whether in the AD or NC groups (Fig 5H). (2) Human Cell_CX3CR1 proportions remained stable across NC and AD groups, while they decreased in AD mice. Cell_APOE proportions were stable in humans but increased in AD mice. Cell_IL1RAPL1 and Cell_SPP1 proportions were consistently higher in humans (Fig 5I). (3) Differences in gene expression patterns were observed between NC and AD groups in both species. The average correlation coefficients for subpopulations were 0.38 and 0.4 between humans and mice (NC: 70–100 years old vs. 21 months old; AD: 70–100 years old vs. 21 months old), with no significant differences (Mann-Whitney-Wilcoxon test, p = 0.61) (Fig 5F, 5J and 5K). These results suggested humans might experience more significant changes from normal progression to AD compared to mice.

### 3.7 Comparison of DEGs related to age or AD between humans and mice

The analysis of genes in DEGs related to age but not AD was conducted. In humans, 217 unique genes(63+53+50+21+8+8+7+2+1×5 = 217) related to age but not AD were identified (p < 0.05, |avg_log2FC| > 0.5) (Fig 3C and S3 Table), with their average expression across disease and age groups displayed in a heatmap (S7A Fig in S1 Appendix). FKBP5 showed age-related changes in microglial subpopulations. KEGG enrichment analyses revealed pathways such as Chagas disease and AGE-RAGE signaling (S7C Fig in S1 Appendix). The average expression level of these genes showed a significant negative correlation with age in the NC group (R = -0.06, P < 0.05), but a non-significant positive correlation in the AD group (R = 0.03, P = 0.40) (S7E Fig in S1 Appendix). In mice, 85 unique genes were identified under the same criteria (Fig 4C and S4 Table), with a heatmap showing their expression across age and disease groups (S7B Fig in S1 Appendix). The expression of mt-Rnr2 was notably higher. KEGG enrichment analyses indicated pathways such as the biosynthesis of amino acids (S7D Fig in S1 Appendix). The average expression of these genes showed non-significant positive correlations with age in both C57BL/6 (R = 0.015, p = 0.78) and APP^_NL-F-G^ groups (R = 0.022, p = 0.68) (S7F Fig in S1 Appendix). Comparative analysis revealed that humans had more age-related genes than mice, with opposite age-related expression trends in the NC group between species but similar trends in the AD group. KEGG pathways were more numerous in humans. Despite differences, a degree of concordance was observed, with 92 DEGs in humans(blue rectangle shape), 29 in mice(red circle shape), and 1 shared gene(yellow diamond shape), indicating some similarity in age-related genes between species (S7G Fig in S1 Appendix).

Similarly, the analysis of genes in DEGs related to AD but not age was performed. In humans, 234 genes(163+32+22+8+6+3 = 234) related to AD but not age were identified (p < 0.05, |avg_log2FC| > 0.5) (Fig 3C), with their average expression across different disease and age groups displayed in a heatmap (S8A Fig in S1 Appendix). The expressions of FRMD4A in Cell_SPP1 and Cell_CX3CR1 were higher in the NC group across all age groups compared to the AD group. KEGG enrichment analyses revealed pathways such as lipid and atherosclerosis, and c-type lectin receptor signaling (S8C Fig in S1 Appendix). The average expression of genes showed a non-significant negative correlation with age in the NC group (R = -0.02, p = 0.08), and a non-significant positive correlation in the AD group (R = 0.02, p = 0.59) (S8E Fig in S1 Appendix). In mice, 185 genes (163+32+22+8+6+3 = 234) related to AD but not age were identified under the same criteria (Fig 4C and S6 Table), with a heatmap showing their expression across disease and age groups (S8B Fig in S1 Appendix). The expression of Ctsd in the Cell_Apoe of the APP^_NL_F_G^ group was higher than in the C57BL/6 group. KEGG enrichment analyses indicated pathways such as cholesterol metabolism and glycerophospholipid metabolism (S8D Fig in S1 Appendix). Average gene expressions showed a non-significant positive correlation with age in the C57BL/6 group (R = 0.001, p = 0.98), and a non-significant negative correlation in the APP^_NL_F_G^ group (R = -0.0009, p = 0.88) (S8F Fig in S1 Appendix). Comparative analysis revealed more AD-related genes in humans than in mice and opposite trends in average gene expression with age between species. However, there was a notable alignment, with 79 genes in humans (blue rectangle shape), 81 in mice (red circle shape), and 5 shared genes(yellow diamond shape), indicating shared aspects of AD-related DEGs irrespective of age between humans and mice (S9A Fig in S1 Appendix).

### 3.8 Comparison of pseudotime analyses for age-related microglial cells in humans and mice

**Fig 6:**
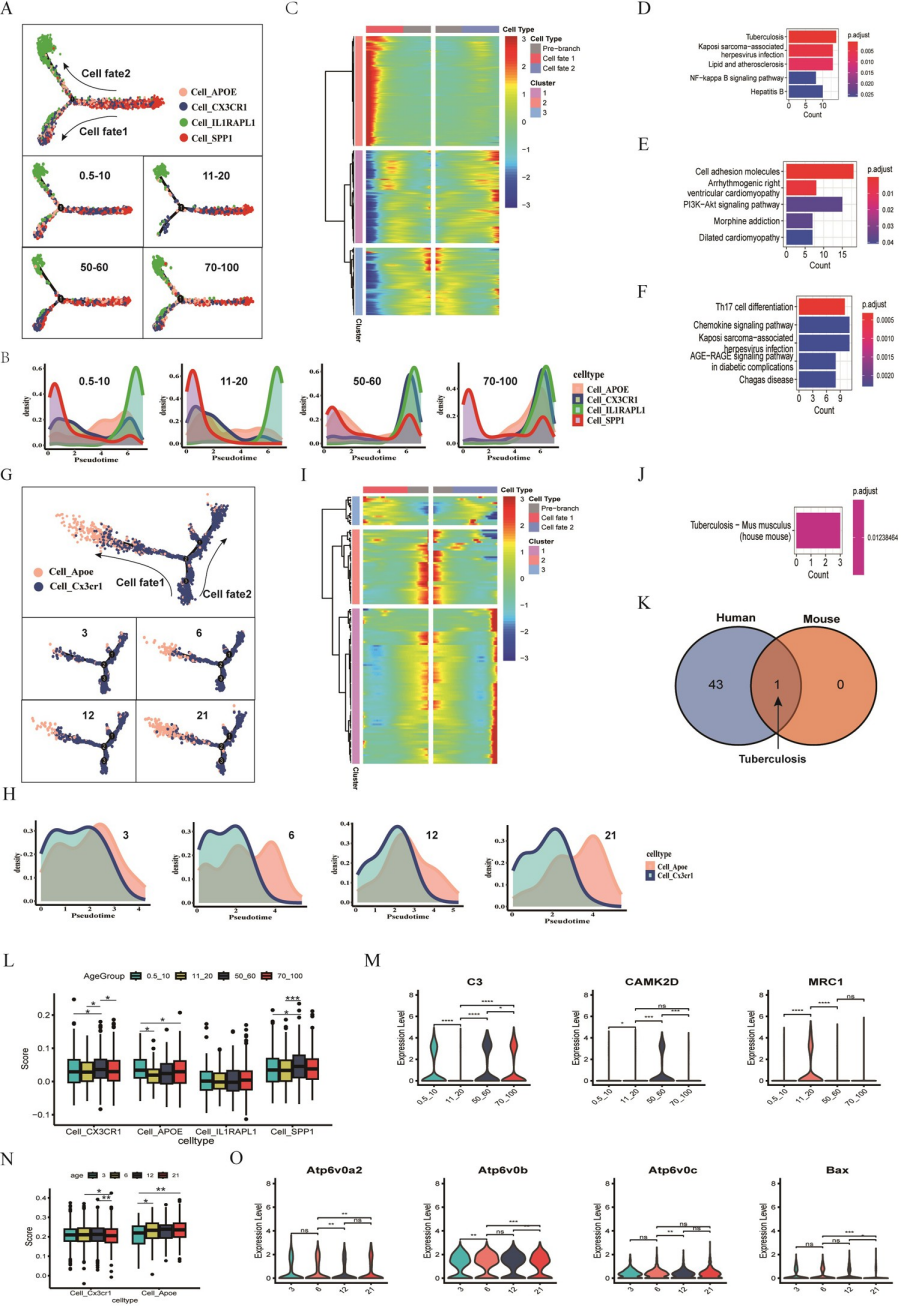
Comparison of pseudotime analyses for age-related microglial cells in humans and mice. A. Age-based distribution and pseudotime analysis of four human microglia subpopulations, showing cell fate differentiation. B. Human microglial cell densities along pseudotime, grouped by age. C. Heatmap of 295 DEGs between young and old humans, divided into three clusters representing cell fate trajectories. D. Top 5 KEGG pathways for cluster 1 genes in the human heatmap. E. Top 5 KEGG pathways for cluster 2 genes in the human heatmap. F. Top 5 KEGG pathways for cluster 3 genes in the human heatmap. G. Age-based distribution and pseudotime analysis of two mouse microglia subpopulations, showing cell fate differentiation. H. Mouse microglial cell densities along pseudotime, grouped by age. I. Heatmap of 108 DEGs between young and old mice, divided into three clusters representing cell fate trajectories. J. KEGG pathways for cluster 2 genes in the mouse heatmap. K. Venn diagram showing the shared pathway (tuberculosis) between humans and mice. Humans had 43 pathways from KEGG enrichment; mice had 1. L. Scoring of human microglial subpopulations using 180 tuberculosis pathway genes, analyzed by age groups with a Mann–Whitney–Wilcoxon test. M. Expression comparison of three genes (C3, CAMK2D, MRC1) in humans across age groups, analyzed with a Mann–Whitney–Wilcoxon test. N. Scoring of mouse microglial subpopulations using 180 tuberculosis pathway genes, analyzed by age groups with a Mann–Whitney–Wilcoxon test. O. Expression comparison of four genes (Atp6v0a2, Atp6v0b, Atp6v0c, Bax) in mice across age groups, analyzed with a Mann–Whitney–Wilcoxon test. Note: Comparisons between groups used two-sided Wilcoxon tests. Asterisks indicated significance levels: * P < 0.05, ** P < 0.01, *** P < 0.001, **** P < 0.0001; NS, not significant.

In our study, to understand age-related changes, we performed pseudotime analyses on microglial cells from both humans and mice. In humans, we identified four subpopulations of microglia and analyzed their distributions across different age groups (Fig 6A). The proportion of Cell_SPP1 was higher in younger individuals (Fig 2C), was chosen as the starting point for pseudotime analysis, leading to two differentiation paths (Fig 6A). We found that Cell_IL1RAPL1 and Cell_SPP1 densities had similar trends across ages, while Cell_CX3CR1 and Cell_APOE showed contrasting patterns (Fig 6B). From human data, we identified 295 DEGs (78 shared in age and AD, 217 age-specific, Fig 3C), grouped into three clusters representing distinct differentiation trajectories (Fig 6C). For mice, pseudotime analysis identified two subpopulations across different ages (Fig 6G). Cell_Apoe had the higher proportion in older mice (Fig 2G), was used as the starting point for pseudotime analysis (Fig 6G). Cell_Cx3cr1 densities remained consistent across ages, while Cell_Apoe densities varied (Fig 6H). We identified 108 DEGs (23 shared in age and AD, 85 age-specific, Fig 4C) for mice, also grouped into three clusters (Fig 6I).

KEGG enrichment analysis identifies pathways for pseudotime heatmap. KEGG enrichment analysis revealed 43 pathways in humans (Fig 6C and S7–S9 Tables), including tuberculosis and Kaposi sarcoma-associated herpesvirus infection (cluster 1), cell adhesion molecules and arrhythmogenic right ventricular cardiomyopathy (cluster 2), and Th17 cell differentiation and chemokine signaling (cluster 3) (Fig 6D–6F). For mice, only the tuberculosis pathway was identified from 3 clusters in the heatmap (Fig 6I and S10 Table), which intriguingly overlapped with the human findings (Fig 6K).

To ascertain the significance of the tuberculosis pathway, a scoring system based on the expression levels of genes in this pathway was applied for both human and mouse microglial subpopulations. Scoring the tuberculosis pathway genes in microglial subpopulations revealed significant differences between age groups in both humans and mice. In humans, significant differences were found in Cell_CX3CR1, Cell_APOE, and Cell_SPP1 across various age groups (Fig 6L). For mice, significant differences were observed in Cell_Cx3cr1 and Cell_Apoe between different age groups (Fig 6N). Additionally, representative genes in the tuberculosis pathway showed significant age-related expression differences in both species (Fig 6M, 6O and S10A Fig in S1 Appendix). These results suggested that there were many differences in the tuberculosis pathway related to age between humans and mice.

In summary, our pseudotime analyses revealed both similarities and differences in age-related changes in microglial cells between humans and mice. Commonalities include similar trends in Cell_APOE/Apoe densities and the shared tuberculosis pathway. Differences include varying trends in Cell_CX3CR1/Cx3cr1 densities across ages between the two species.

### 3.9 Comparison of pseudotime analyses for AD-related microglial cells in humans and mice

**Fig 7:**
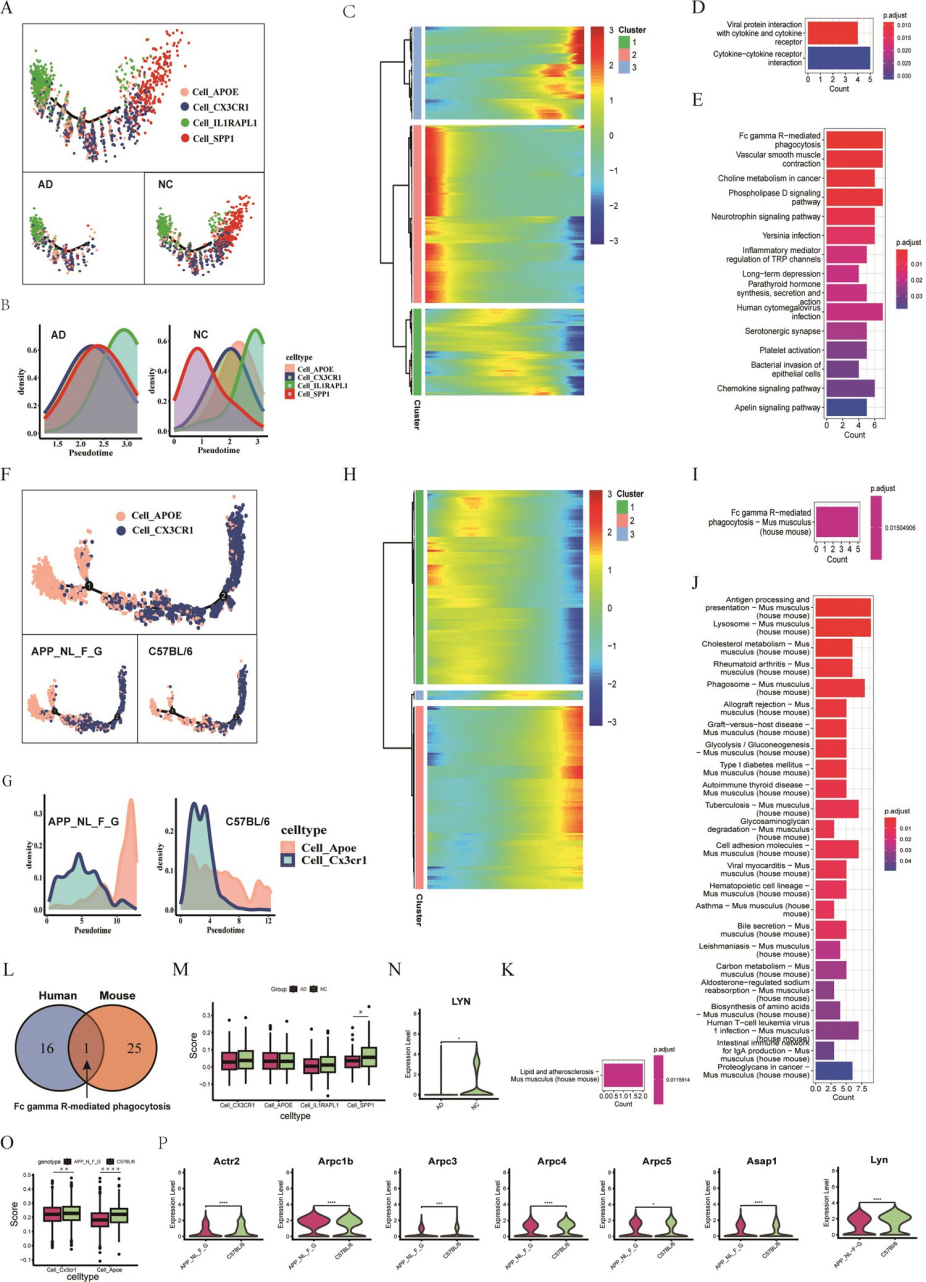
Comparison of pseudotime analyses for AD-related microglial cells in humans and mice. A. Distribution of four human microglial subpopulations by disease status, showing cell differentiation using pseudotime analysis. B. Human microglial cell densities along pseudotime, grouped by NC and AD status. C. Heatmap of the top 312 DEGs between NC and AD groups in humans, divided into three clusters with differentiation trajectories. D. Two KEGG pathways identified for cluster 1 genes in the human heatmap. E. Top 15 KEGG pathways identified for cluster 2 genes in the human heatmap. F. Distribution of two mouse microglial subpopulations by NC and AD groups, showing cell differentiation using pseudotime analysis. G. Mouse microglial cell densities along pseudotime, grouped by NC and AD status. H. Heatmap of the top 208 DEGs between NC and AD groups in mice, divided into three clusters with differentiation trajectories. I. KEGG pathways identified for cluster 1 genes in the mouse heatmap. J. KEGG pathways identified for cluster 2 genes in the mouse heatmap. K. KEGG pathways identified for cluster 3 genes in the mouse heatmap. L. Venn diagram showing shared pathways between humans and mice. 17 pathways were identified from the human heatmap clusters, and 26 pathways from the mouse heatmap clusters. M. Scores of four human microglial subpopulations using 97 genes in the Fc gamma R-mediated phagocytosis pathway, by NC and AD status. N. Expression comparison of the LYN gene in humans between NC and AD groups. O. Scores of two mouse microglial subpopulations using 93 genes in the Fc gamma R-mediated phagocytosis pathway, by APP^_NL_F_G^ and C57BL/6 groups. P. Expression comparison of seven genes (Actr2, Arpc1b, Arpc3, Arpc4, Arpc5, Asap1, and Lyn) in mice between APP^_NL_F_G^ and C57BL/6 groups. Note: Two-sided Wilcoxon tests were used for comparisons between groups. Significance levels: * P < 0.05, ** P < 0.01, *** P < 0.001, **** P < 0.0001; NS, not significant.

We performed pseudotime analyses on microglial cells related to AD in both humans and mice. For human subjects, we identified four microglial subpopulations, grouped by disease status (Fig 7A). The Cell_SPP1 subpopulation decreased in the AD group compared to the NC group. Cell densities along pseudotime for Cell_CX3CR1, Cell_APOE, and Cell_IL1RAPL1 showed consistent trends, whereas Cell_SPP1 densities differed between AD and NC groups (Fig 7B). We identified 312 DEGs related to age and AD (78 shared, 234 AD-specific, Fig 3C), clustered into three groups with similar kinetic trends (Fig 7C). For mice, two microglial subpopulations were identified, divided by disease status (Fig 7F). Cell_Apoe was more distribution in the APP^_NL_F_G^ group compared to the C57BL/6 group (Fig 7G). Cell_Cx3cr1 densities were consistent between groups, while Cell_Apoe densities varied. We identified 208 DEGs related to AD (23 shared, 185 AD-specific, Fig 4C), also clustered into three groups with similar kinetic trends (Fig 7H).

KEGG enrichment analysis provided insights into pathways linked to pseudotime heatmap clusters. In humans, two pathways (viral protein interaction with cytokine and cytokine receptor, cytokine-cytokine receptor interaction) were identified for cluster 1 (Fig 7D and S11 Table), two pathways (neurotrophin signaling, Fc gamma R-mediated phagocytosis) for cluster 2 (Fig 7E and S12 Table), and no pathways for cluster 3. In mice, Fc gamma R-mediated phagocytosis was identified for cluster 1 (Fig 7I and S13 Table), antigen processing and presentation, lysosome pathways for cluster 2 (Fig 7J and S14 Table), and lipid and atherosclerosis for cluster 3 (Fig 7K and S15 Table). Humans had 17 unique pathways (Fig 7C and S11 and S12 Tables), and mice had 26 unique pathways (Fig 7H and S13–S15 Tables). Fc gamma R-mediated phagocytosis was a shared pathway between species (Fig 7L).

The comparison of the Fc gamma R-mediated phagocytosis pathway revealed differences between species. In humans, 97 pathway genes were used to score microglial subpopulations, showing significant differences in Cell_SPP1, with lower expression of LYN in the AD group (Fig 7M and 7N). In mice, 93 pathway genes indicated significant differences in Cell_Cx3cr1 and Cell_Apoe (p<0.01, p<0.0001, Fig 7O), and 24 representative genes showed significant expression differences between APP^_NL_F_G^ and C57BL/6 groups (p<0.05, Fig 7P, S10B Fig in S1 Appendix).

In summary, pseudotime analyses of AD-related microglial cells in humans and mice revealed similarities and differences.(1) Similarities: Cell_APOE/Apoe densities had similar trends in both species, and the Fc gamma R-mediated phagocytosis pathway was shared between humans and mice.(2) Differences: Cell_CX3CR1/Cx3cr1 densities showed different trends between humans and mice.

## 4 Discussion

This paper investigated similarities and differences in microglia between humans and mice at scRNA-seq level, related to age and AD: **(1) Similarities:** The proportion of microglial subpopulation Cell_APOE/Apoe exhibited a consistent trend in both AD and NC groups in humans and mice. The proportion of Cell_CX3CR1/Cx3cr1 as homeostatic microglia remained stable with age in NC groups of both species. The tuberculosis pathway was commonly engaged in the response of microglia to age in both humans and mice. Similarly, the Fc gamma R-mediated phagocytosis pathway was shared in the response of microglia to AD in humans and mice. **(2) Differences:** IL1RAPL1 and SPP1 as marker genes were easier to identify microglia subpopulations in humans compared to Il1rapl1 and Spp1 in mice. The majority of DEGs associated with age or AD changed with age. These changes exhibited different trends between humans and mice. Pseudotime analyses demonstrated varying trends in cell density for microglial subpopulations based on age or AD between humans and mice.

### 4.1 Translational potential: Replacing human research with mouse models

The APOE gene, which is involved in lipid metabolism and serves as a genetic risk factor for AD) [10, 20], has been expressed in microglia in both humans and mice. High expression of APOE in both species has been shown to lead to AD by altering microglial function [21, 22]. Despite this, humans possess distinct APOE alleles (APOE2, APOE3, APOE4) that play different roles in AD [20], whereas mice have only one allele. This discrepancy has resulted in different impacts of APOE on microglia between the two species. In our study, we observed divergent trends in the cell densities of APOE-labeled microglia along pseudotime between humans and mice in both AD and NC groups. Additionally, we found that in humans, the number of down-regulated genes in APOE-labeled microglia was fewer than the number of up-regulated genes, which was the opposite trend observed in mice. This suggests that although mice can be beneficial for studying APOE’s role in AD, the underlying mechanisms may differ from those in humans, necessitating cautious interpretation.

CX3CR1/Cx3cr1 was a marker for homeostatic microglia in both species [9]. The disruption of homeostasis is critical in the processes of brain aging and neurodegeneration. Our research demonstrated a significant reduction in Cx3cr1-labeled microglia in AD model mice (APP^_NL_F_G^) when compared to control conditions, whereas CX3CR1-labeled microglia remained stable in both normal elderly humans and AD patients. This highlighted potential differences in microglial responses to AD between humans and mice. Moreover, the risk genes for AD, IL1RAPL1 and SPP1, which play roles in synaptic regulation [23, 24], might be able to identify specific microglial subpopulations in humans. However, they did not do so in mice, indicating significant differences in microglial subpopulations between these two species. APP^_NL_F_G^ mice, which were employed as AD models, might not be suitable for human studies focusing on the roles of IL1RAPL1 and SPP1 genes in AD.

At the genetic level, age- and AD-related DEGs in microglia differ greatly between humans and mice, with only SPP1 overlapping. DEGs associated with age exhibit a positive correlation with age in both the human AD group and mouse models (APP^_NL_F_G^, C57BL/6). In contrast, these DEGs are negatively correlated with age in normal human subjects. Furthermore, DEGs related to AD show a negative correlation with age in the NC group of humans and mouse models (APP^_NL_F_G^), while they are positively correlated with age in the human AD group and the mouse C57BL/6 group. This contrasting behavior of DEGs in different contexts highlights the complexity of age-related gene expression in AD. These findings emphasize the need to carefully consider age as a variable in both human and animal studies of AD, as it may influence the interpretation of gene expression data and the understanding of disease mechanisms. Hence, caution is necessary when using mouse models as substitutes for human samples in research on the regulation of AD by microglia.

### 4.2 Applications of the mouse models in the specific pathway research

Our pseudotime analysis has identified shared pathways that hold promise for cross-species studies. Specifically, the tuberculosis pathway and the Fc gamma R-mediated phagocytosis pathway are shared between humans and mice. The tuberculosis pathway, relevant to age-related studies, is linked to Mycobacterium tuberculosis (Mtb), which inhabits phagocytic cells in macrophages. Mtb can evade the immune system, activate latent tuberculosis, and impact various immune responses such as phagosome maturation, T cell exhaustion, altered antigen expression, cell trafficking, and transmission [25–27]. The Fc gamma R-mediated phagocytosis pathway has emerged as a potential focus for AD research [28]. Fc gamma receptors (FcγR) binding to the Fc portion of IgG are crucial in immune responses, facilitating the clearance of Aβ plaques by microglia in both species [29]. Dysregulation of Fcγ receptor-mediated phagocytosis is associated with AD pathophysiology [30]. Both pathways are associated with immune responses and microglial phagocytosis. Our findings suggest that the tuberculosis pathway’s association with immune responses and age-related factors, and the Fc gamma R-mediated phagocytosis pathway’s link to AD pathology, make them suitable for cross-species studies focused on aging and AD.

### 4.3 Novel marker genes related to age and AD

In this study, aside from uncovering the similarities and differences in the mechanisms by microglia regulating AD in both mouse models and human patients, as well as the aging process in normal humans and mice, we also identified several novel genes that might be related to the regulatory role of microglia in AD and aging. We pinpointed thirteen genes initially associated with aging and AD, including five novel genes (RP11-557H15.4, CHKA, SNHG5, DCAF6, INPP4B) related to age and AD in humans, and eight novel genes (Lyz2, Gpr165, Upk1b, Cc16, CD9, Lilrb4a, H2-Ab1, Itgax) in mice. These genes had not been previously linked to aging and AD in the literature. Additionally, four genes (Axl, CAPG, HIF1α, APLP2, and PDCD1) were reported for their roles in microglia or AD. Upregulation of Axl enhanced microglial phagocytosis [31], while CAPG served as a marker for microglial activation [32]. HIF1α potentially regulated synaptosome phagocytosis [33]. Although APLP2 and PDCD1 were not directly connected to aging and AD, their gene families were implicated in AD [34–36], and PDCD1 regulated immune responses [36]. These findings suggest that these genes might play roles in the mediation of AD and the regulation of aging by microglia. Conducting further research in this area can help us to broaden our understanding of the mechanisms by which microglia regulated AD and aging.

### 4.4 Challenges and issues

In summary, we find that some microglial changes are consistent between AD mouse models and human AD patients; however, there are also several distinct differences. These differences are particularly pronounced within specific microglial subpopulations defined by certain genes, such as the microglial subpopulations identified APOE, CX3CR1, IL1RAPL1 and SPP1. Additionally, there are significant differences in microglia-associated genes between AD mouse models and human AD patients. Therefore, caution is necessary when using AD mouse models for microglial studies. These models are generally suitable for examining the overall role of microglia in AD, but they may not be appropriate for research focused on specific microglial subpopulations or gene-level investigations. To enhance our understanding of the role of specific microglial subpopulations in AD, it is essential to conduct comparative studies across various model organisms to identify the most appropriate models. Alternatively, developing mouse models with specific microglial phenotypes through targeted gene editing of microglia-associated genes would provide a more accurate platform for AD research. These approaches could offer a clearer insight into the functions of distinct microglial subpopulations in AD.

### 4.5 Limitations

The study integrated the snRNA-seq dataset GSE198323 from human hippocampal microglia and the scRNA-seq dataset GSE127892 from mouse hippocampal microglia. These datasets were merged using the ‘harmony method’ from the Seurat package in R. Although this approach has been used in many different research [37, 38], using the same data type might be more accurate. In this research, the microglia were extracted from the human hippocampus sample data to compare with mouse microglia, which might introduce some unknown errors. Additionally, while 3rd generation AD models (App^_NL_F^ and Psen1P117L/WT) exist, no single-cell datasets are available, so 2nd generation AD model mice (App^_NL_G_F^) were used instead. Therefore, future research should involve selecting more appropriate samples, increasing sample sizes, and conducting additional experiments to enhance the reliability of the findings.

### 4.6 Conclusions

In conclusion, our research highlights the utility and limitations of mice as models for studying AD at the single-cell level, revealing both conserved and species-specific markers. These insights inform more judicious model selection and experimental design in the quest to understand the molecular bases of aging and AD across species.

## Supporting information

S1 Appendix Supplementary materials. (DOCX)

S1 Table Summary of human specimens used in the study. (XLSX)

S2 Table 78 shared genes in DEGs related to age and AD. (XLSX)

S3 Table 217 shared genes in DEGs related to human age. (XLSX)

S4 Table 85 shared genes in DEGs related to mouse age. (XLSX)

S5 Table 234 shared genes in DEGs related to human AD. (XLSX)

S6 Table 185 shared genes in DEGs related to mouse AD. (XLSX)

S7 Table Pathways from KEGG enrichment analysis with genes of cluster1 in the heatmap for humans (Fig 6C). (XLSX)

S8 Table Pathways from KEGG enrichment analysis with genes of cluster2 in the heatmap for humans (Fig 6C). (XLSX)

S9 Table Pathways from KEGG enrichment analysis with genes of cluster3 in the heatmap for humans (Fig 6C). (XLSX)

S10 Table Pathways from KEGG enrichment analysis with genes of cluster2 in the heatmap for mice (Fig 6I). (XLSX)

S11 Table Pathways from KEGG enrichment analysis with genes of cluster1 in the heatmap for humans (Fig 7C). (XLSX)

S12 Table Pathways from KEGG enrichment analysis with genes of cluster2 in the heatmap for humans (Fig 7C). (XLSX)

S13 Table Pathways from KEGG enrichment analysis with genes of cluster1 in the heatmap for mice (Fig 7H). (XLSX)

S14 Table Pathways from KEGG enrichment analysis with genes of cluster2 in the heatmap for mice (Fig 7H). (XLSX)

S15 Table Pathways from KEGG enrichment analysis with genes of cluster3 in the heatmap for mice (Fig 7H). (XLSX)
