# Supplementary material for: Exploring the feasibility of using mice as a substitute model for investigating microglia in aging and Alzheimer’s disease though single cell analysis
Source: PLoS One. 2024 Nov 26;19(11):e0311374. doi: 10.1371/journal.pone.0311374 (PMC11594518; doi:10.1371/journal.pone.0311374)
Supplement: S1 Appendix — (DOCX) [file pone.0311374.s001.docx]

# Supplementary Materials

## Materials and methods

### Data downloaded

GSE198323 ^[1]^ and GSE127892^[2]^ were downloaded from Gene Expression Omnibus (GEO) database of NCBI (https://www.ncbi.nlm.nih.gov/). GSE198323 was a snRNA-seq dataset of human hippocampus including 23 normal control(NC) and 8 alzheimer's disease (AD) samples with age ranging from 0.6 to 92 years old (Table S1).Moreover, the 31samples were divided into four groups by age:0.5_10,11_20,50_60 and 70_100. GSE127892 was another single cell RNA-seq dataset of microglial cells in mouse hippocampus including C57Bl/6 wild-type and AppNL-G-F mice, which had four different time points: 3,6,12,21 months old.

### Quality control

Datasets of GSE198323 and GSE127892 were created independently seurat object by the Seurat R package (version 4.0, <https://satijalab.org/seurat/>) for downstream analysis, respectively. Before quality control, the number of mRNAs, the reads of mRNA and the percentage of mitochondrial genes for human (GSE198323) grouped by disease were visualized in violin plots (Fig. S1A, S1B, S1C). The number of mRNAs, the reads of mRNA and the percentage of mitochondrial genes for mouse grouped by disease were visualized in violin plots (Fig. S1G, S1H, S1I). When quality control was performed, for human (GSE198323), the UMI counts of each sample were discarded with either more than 3500 genes or less than 200 genes, and mitochondrial genes percentage more than 5%. Filtered data included 32614 genes and 124119 cells. After quality control, the number of mRNAs, the reads of mRNA and the percentage of mitochondrial genes for human grouped by disease were visualized in violin plots (Fig. S1D, S1E, S1F). For GSE127892, cells in UMI counts were discarded with either more than 3500 genes or less than 200 genes, and mitochondrial genes percentage more than 5%. Filtered data included 29025 genes and 10689 cells. After quality control, the number of mRNAs, the reads of mRNA and the percentage of mitochondrial genes for human grouped by disease were visualized in violin plots (Fig. S1K, S1K, S1L).

### 3. Cell type clustering and cell type identification

*NormalizeData* and *ScaleData* function in Seurat R package were applied for filtered data to generate normalize expression matrices. *RunPCA* function was used to identify significant principal components (PCs). Harmony as a batch effect correction algorithm (<https://github.com/immunogenomics/harmony>) was performed to correct heterogeneity of samples. *FindClusters* function with resolution 1.0 was performed to obtain 22 clusters for human dataset (GSE198323), and 14 clusters for mouse dataset(GSE127892), respectively. Then, uniform manifold approximation and projection (UMAP) and t-distributed stochastic neighbor embedding (t-SNE) were applied to exhibited subpopulations of human hippocampus, which were manually annotated by canonical marker genes reported in previous studies ^[3,4,5,6]^. The canonical marker genes as follows: astrocytes (AQP4), endothelial cells (FLT1), microglia (APBB1IP), oligodendrocytes (MOBP), oligodendrocyte progenitor cells (VCAN), dentate gyrus neurons (PROX1), pyramidal neurons (TSHZ2), inhibitory neurons (GAD2), and subtype of excitatory neurons (ExN.sub). SYT1 was marker gene of neurons, and NEUROD2 was marker gene of excitatory neurons, so we identified the excitatory neurons except for dentate gyrus neurons and pyramidal neurons as ExN.sub. Subsequently, microglia cells of human hippocampus (GSE198323) were extracted by using the *Subset* function, which would be compared with microglia cells of mouse hippocampus based on age and disease (AD).

### Sub-cluster analysis

After data normalization and scaling were performed for microglia cells of humans and mice, respectively, the principal component analysis (PCA) was used to analyze and identify significant principal components. Subsequently, the *FindClusters* function with the parameters resolution = 1 was used to classify cells into different clusters and UMAP plots were applied to visualized subpopulations of microglia cells. For microglia cells of human, CX3CR1 and APOE as known marker genes of homeostatic and DAM-like microglia reported in previous studies ^[7]^ were used to identify Cell_CX3CR1 and Cell_APOE subpopulations, respectively (Fig.S6A). In addition, several genes with high expression were identified by *FindAllMarkers* function as marker genes (IL1RAPL1/Il1rapl1 and SPP1/Spp1) of the other subpopulations including Cell_ IL1RAPL1 and Cell_SPP1 for human microglia cells, respectively (Fig.S6A). Cx3cr1 and Apoe as known marker genes of homeostatic and DAM-like microglia reported in previous studies ^[7]^ were used to identify Cell_Cx3cr1 and Cell_Apoe subpopulations for mice, respectively (Fig.S6C).

### Differentially expressed genes analysis

Differentially expressed genes (DEGs) analysis was performed involved in age and disease (AD). Firstly, DEGs involved in age were analyzed. For humans(GSE198323), the samples belonged to NC group and age in 0.5_10 and 11-20 age groups were selected as young group, and the samples belonged to NC group and age in 70_100 age group were selected as old group. For mice (GSE127892), the samples belonged to C57Bl/6 group and age in 3 and 6 month old were selected as young group, and the samples belonged to C57Bl/6 group and age in 21 month old were selected as old group. Next, the *FindAllMarkers* function was used to find DEGs in each subpopulation of microglia between young and old groups with MAST test with | log fold change| ≥0.5 and p_val_adj<0.05 for humans and mice, respectively. Secondly, DEGs involved in AD were analyzed. For humans, the samples belonged to NC group and age in 70_100 age group were selected as NC group, and the samples belonged to AD group and age in 70_100 age group were selected as AD group. For mice, the samples belonged to C57Bl/6 group and age in 12 and 21 month old were selected as NC group, and the samples belong to APP_NL.F.G group and age in 12 and 21 month old were selected as AD group. Next, the *FindAllMarkers* function was used to find DEGs in each subpopulation of microglia between NC and AD groups with MAST test with | log fold change| ≥0.5 and p_val_adj<0.05 for humans and mice, respectively.

Genes shared in DEGs involved with age and DEGs involved with disease, unique genes in DEGs related to age and not to AD, and unique genes in DEGs related to disease (AD), not to age were selected by upset plot for humans and venn diagram for mice, respectively. Then, Genes shared in DEGs involved with age and DEGs involved with disease, unique genes in DEGs related to age and not to AD, and unique genes in DEGs related to disease (AD), not to age were visualized by heatmaps for humans and mice, respectively. The average expressions of genes of these DEGs were displayed by a scatterplot with linearly fitted curve to observe the expression change with age, respectively. KEGG enrichment analyses were performed with genes in these DEGs, respectively. In order to observed that the expressions of genes shared in DEGs involved with age and disease varied with age, we obtained less genes by using | log fold change| ≥1 and p_val_adj<0.05 for human and mice, respectively. Finally, the expressions of these genes were exhibited by using dotplots and scatterplots, respectively.

Similarities and differences of genes between humans and mice for microglial cells were compared. Gene sets compared included as follow: genes shared in DEGs involved with age and DEGs involved with disease, unique genes in DEGs related to age and not to AD, and unique genes in DEGs related to disease (AD), not to age.

### 6. PPI Network by using DEGs

(1) To investigate the relation of 78 genes of DEGs of humans and 23 genes of DEGs of mice shared in DEGs related with age and DEGs related with disease (AD), we created PPI Network by using STRING database and Cytoscape. Firstly, homologene package in R was used to convert these 23 genes of mouse into 20 homologous genes in human. Secondly, 97 genes were obtained by calculating the union of 20 homologous genes and 78 genes in human. Finally, these 97 genes were submitted to STRING database to obtain PPI data, and a protein-protein interaction (PPI) network was constructed by Cytoscape 3.9.0(Fig.3I). (2) To investigate the relation of the 217 genes of DEGs of human related with age and not related with AD and 85 genes of DEGs of mouse related with age and not related with AD, we created PPI Network by using the same method. Firstly, 85 genes of DEGs of mouse were converted into 74 homologous genes in human. Secondly, 288 genes were obtained by calculating the union of 74 homologous genes and 217 genes in human. Finally, these 288 genes were submitted to STRING database to obtain PPI data, and a protein-protein interaction (PPI) network was constructed by Cytoscape 3.9.0 (Fig.S13G). (3) To investigate the relation of the 234 genes of DEGs of human related with AD and not related with age and 185 genes of DEGs of mouse s related with AD and not related with age, we created PPI Network by using the same method. Firstly, 185 genes of DEGs of mouse were converted into 164 homologous genes in human. Secondly, 389 genes were obtained by calculating the union of 164 homologous genes and 234 genes in human. Finally, these 389 genes were submitted to STRING database to obtain PPI data, and a protein-protein interaction (PPI) network was constructed by Cytoscape 3.9.0 (Fig.S15A).

### 7. Cross-species analysis in humans and mice

Firstly, we only used the one-by-one orthologous genes (n = 14034) of humans and mice for cross-species comparison analysis. The cell count matrices of the orthologous genes of microglial cells were extracted from scRNA-seq data of humans (GSE198323) and mice (GSE127892), respectively. The scRNA-seq data of humans and mice were integrated by using the Seurat package. Harmony as a batch effect correction algorithm (<https://github.com/immunogenomics/harmony>) was performed to correct heterogeneity of samples. Then, Clustering cell, cell-type annotation and marker genes were performed as described above. The similarity of cells for cross-species was measured by using the spearman’s rank correlations of average expression values of orthologous genes, which were calculated in humans and mice for each cell type at scRNA-seq level. The spearman’s rank correlations were displayed in heatmaps by using pheatmap package of R. Finally, the spearman’s rank correlations were compared by using boxplot.

### 8. Pseudotime analysis

Pseudotime analysis was performed by using Monocle2 (version 2.99.3) as a R package. A *CellDataSet* object was created from Seurat analysis. *DDRTree* method and *orderCells* function were used for dimensional reduction and ordering cell, respectively. Then, the pathways shared in humans and mice were calculated by using heatmaps inferred from Monocle2. The gene set of pathway of KEGG for humans or mice was downloaded from Kyoto Encyclopedia of Genes and Genomes database (KEGG, https:// www.genome.jp/kegg/). The *AddModuleScore* function of the Seurat R package was used to calculate scores for gene expression of gene set group by age groups or disease. The scores of AD group compared with that of NC group by using a Mann–Whitney–Wilcoxon ranked test. The scores were compared in differently paired age groups by using a Mann–Whitney–Wilcoxon ranked test. The expression levels of genes in pathway were calculated between AD and NC groups or different age groups by using a Mann–Whitney–Wilcoxon ranked test with statistically significant (p<0.05) displayed by violin plot.

### Reference

1. Zhou Y, Su Y, Li S, Kennedy BC, Zhang DY, Bond AM, Sun Y, Jacob F, Lu L, Hu P, Viaene AN, Helbig I, Kessler SK, Lucas T, Salinas RD, Gu X, Chen HI, Wu H, Kleinman JE, Hyde TM, Nauen DW, Weinberger DR, Ming GL, Song H. Molecular landscapes of human hippocampal immature neurons across lifespan. Nature. 2022 Jul;607(7919):527-533.
2. Sala Frigerio C, Wolfs L, Fattorelli N, Thrupp N, Voytyuk I, Schmidt I, Mancuso R, Chen WT, Woodbury ME, Srivastava G, Möller T, Hudry E, Das S, Saido T, Karran E, Hyman B, Perry VH, Fiers M, De Strooper B. The Major Risk Factors for Alzheimer's Disease: Age, Sex, and Genes Modulate the Microglia Response to Aβ Plaques. Cell Rep. 2019 Apr 23;27(4):1293-1306.e6.
3. Ayhan F, Kulkarni A, Berto S, Sivaprakasam K, Douglas C, Lega BC, Konopka G. Resolving cellular and molecular diversity along the hippocampal anterior-to-posterior axis in humans. Neuron. 2021 Jul 7;109(13):2091-2105.e6.
4. Arneson D, Zhang G, Ying Z, Zhuang Y, Byun HR, Ahn IS, Gomez-Pinilla F, Yang X. Single cell molecular alterations reveal target cells and pathways of concussive brain injury. Nat Commun. 2018 Sep 25;9(1):3894.
5. Zhong S, Ding W, Sun L, Lu Y, Dong H, Fan X, Liu Z, Chen R, Zhang S, Ma Q, Tang F, Wu Q, Wang X. Decoding the development of the human hippocampus. Nature. 2020 Jan;577(7791):531-536.
6. Hochgerner H, Zeisel A, Lönnerberg P, Linnarsson S. Conserved properties of dentate gyrus neurogenesis across postnatal development revealed by single-cell RNA sequencing. Nat Neurosci. 2018 Feb;21(2):290-299.
7. Chen Y, Colonna M. Microglia in Alzheimer's disease at single-cell level. Are there common patterns in humans and mice? J Exp Med. 2021 Sep 6;218(9):e20202717.

### Figure S1


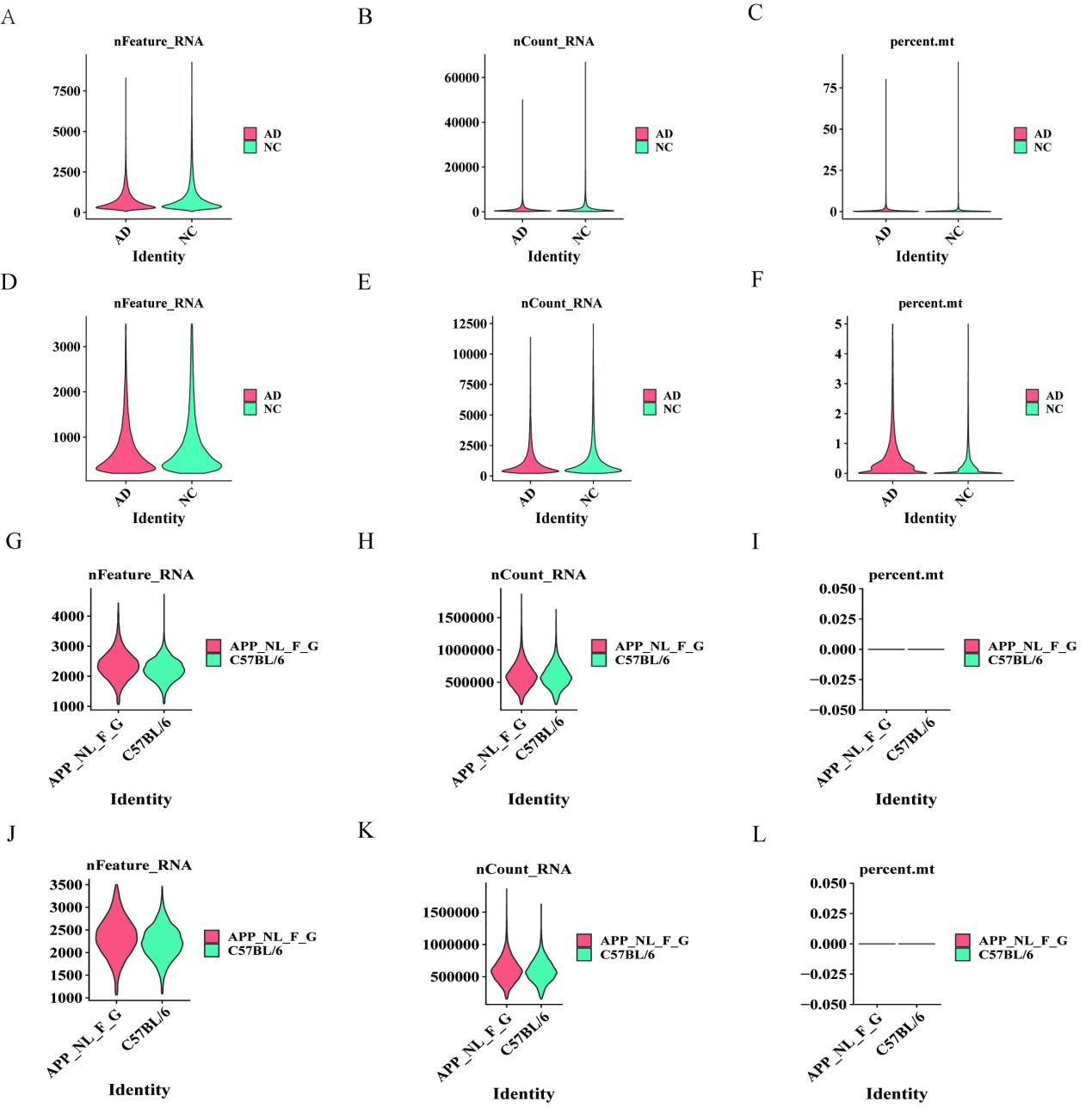


Figure S1 Quality control for humans (GSE198323) and mice (GSE127892).

1. Violin plot of the number of mRNAs group by disease for GSE198323 before filtered.
2. Violin plot of the reads of mRNA group by disease for GSE198323 before filtered.
3. Violin plot of the percentage of mitochondrial genes group by disease for GSE198323 before filtered.
4. Violin plot of the number of mRNAs group by disease for GSE198323 after filtered.
5. Violin plot of the reads of mRNA group by disease for GSE198323 after filtered.
6. Violin plot of the percentage of mitochondrial genes group by disease for GSE198323 after filtered.
7. Violin plot of the number of mRNAs group by disease for GSE127892 before filtered.
8. Violin plot of the reads of mRNA group by disease for GSE127892 before filtered.
9. Violin plot of the percentage of mitochondrial genes group by disease for GSE127892 before filtered.
10. Violin plot of the number of mRNAs group by disease for GSE127892 after filtered.
11. Violin plot of the reads of mRNA group by disease for GSE127892 after filtered.
12. Violin plot of the percentage of mitochondrial genes group by disease for GSE127892 after filtered.

### Figure S2


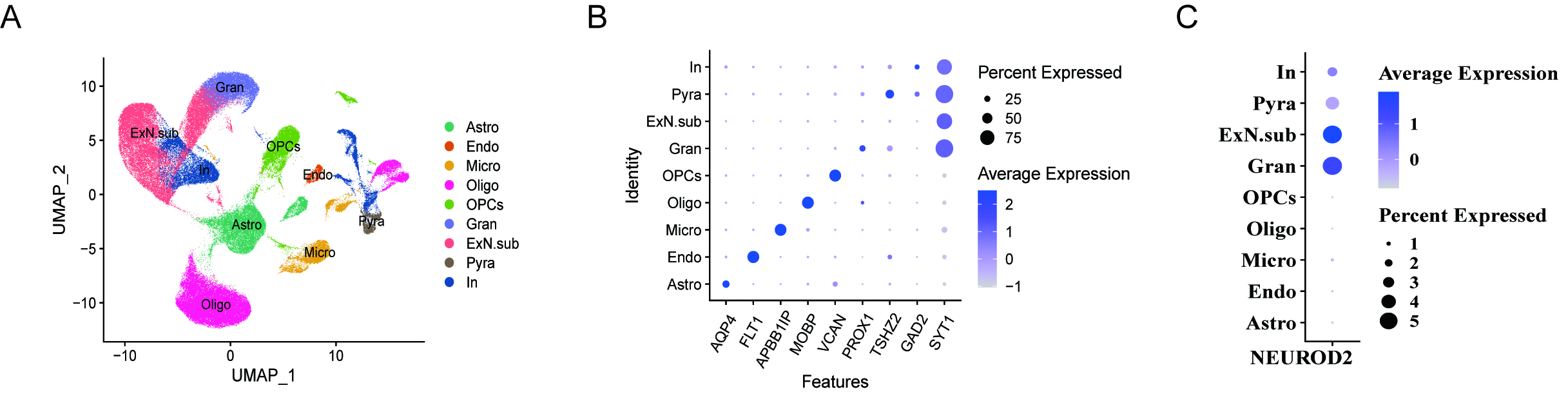


Figure S2 Cell type identification for humans (GSE198323).

1. Distributions of 9 major subpopulations of hippocampus for human (GSE198323) by using an UMAP plot.
2. A dotplot exhibited the expression for marker genes for 9 major subpopulations of hippocampus for human (GSE198323). The canonical marker genes as follows: astrocytes (AQP4), endothelial cells (FLT1), microglia (APBB1IP), oligodendrocytes (MOBP), oligodendrocyte progenitor cells (VCAN), dentate gyrus neurons (PROX1), pyramidal neurons (TSHZ2), inhibitory neurons (GAD2), and subtype of excitatory neurons (ExN.sub). SYT1 was marker gene of neurons.
3. A dotplot exhibited the expression for NEUROD2 as marker gene for excitatory neurons of hippocampus for human (GSE198323).

### Figure S3


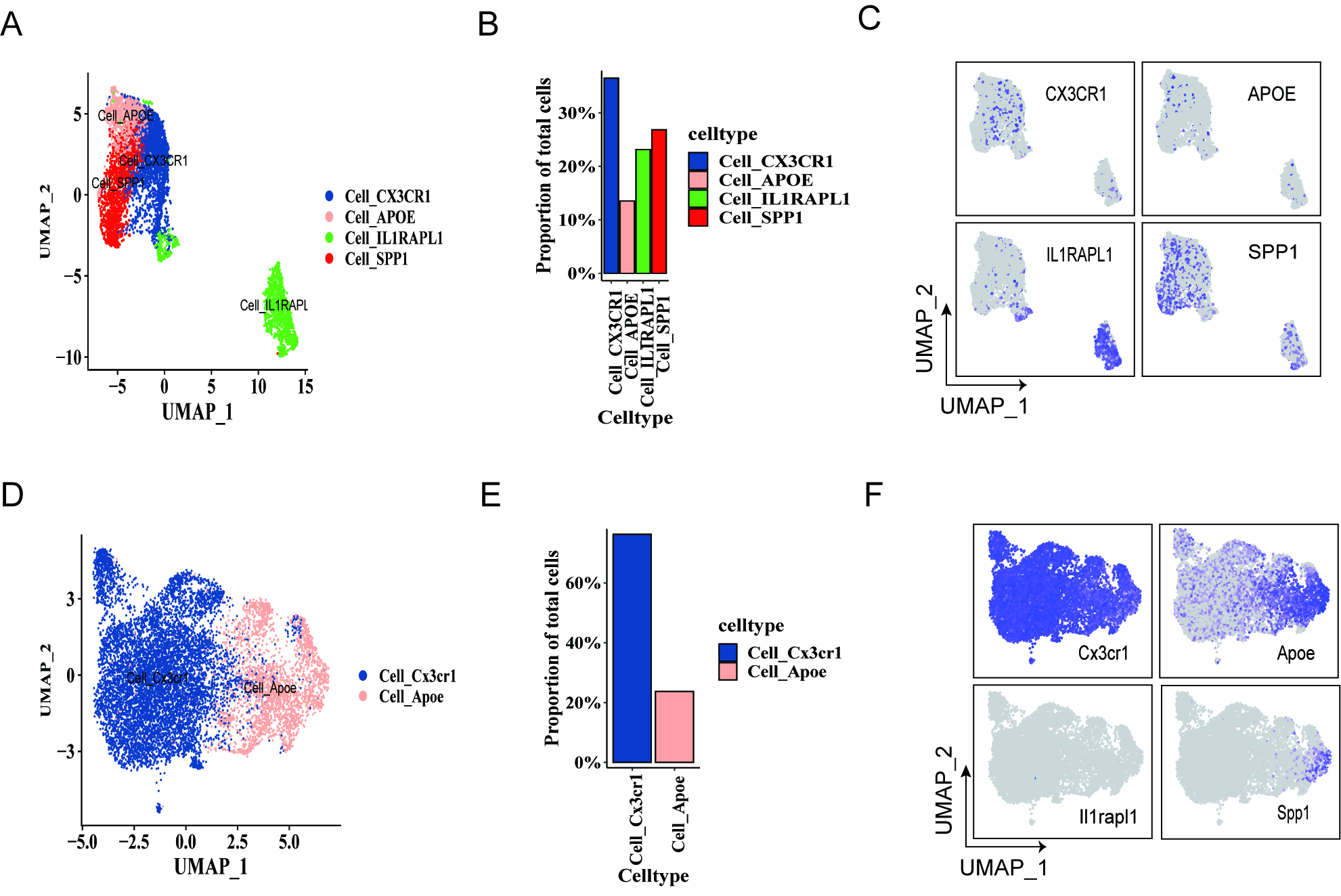


Figure S3 Subpopulations of microglia identification for humans (GSE198323) and mice (GSE127892).

1. Distributions of 4 major subpopulations of microglia for humans (GSE198323) by using an UMAP plot. 4 major subpopulations included Cell_ CX3CR1, Cell_APOE, Cell_IL1RAPL1 and Cell_SPP1.
2. The percentage of 4 major subpopulations of microglia for humans (GSE198323).
3. Marker gene (CX3CR1, APOE, IL1RAPL1, SPP1) distributions for these subpopulations in human microglia.
4. Distributions of 2 major subpopulations of microglia for mice (GSE127892) by using an UMAP plot. 2 major subpopulations included Cell_ Cx3cr1, Cell_Apoe, Cell_Il1rapl1 and Cell_Spp1.
5. The percentage of 2 major subpopulations of microglia for mice (GSE127892).
6. Marker gene (Cx3cr1, Apoe) distributions for these subpopulations, and Il1rapl1 and Spp1 distributions in mouse microglia.

### Figure S4


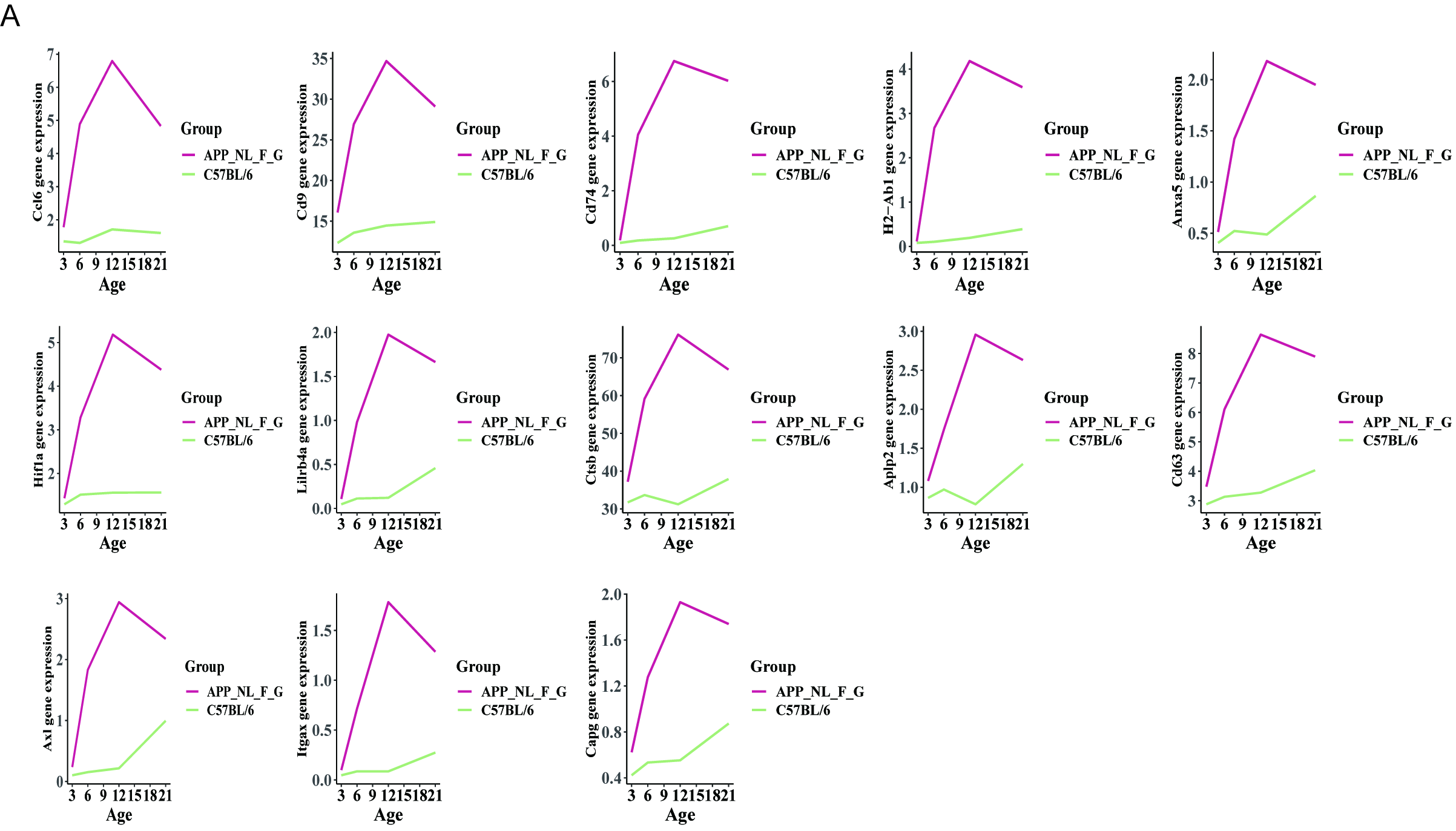


Figure S4 The comparison of the average expressions of 13 genes between C57Bl/6 and APP_NL.F.G groups, these genes shared in DEGs related with age and DEGs related with disease.

1. The comparison of the average expressions of 13 genes between C57Bl/6 and APP_NL.F.G groups, these genes shared in DEGs related with age and DEGs related with disease.

### Figure S5


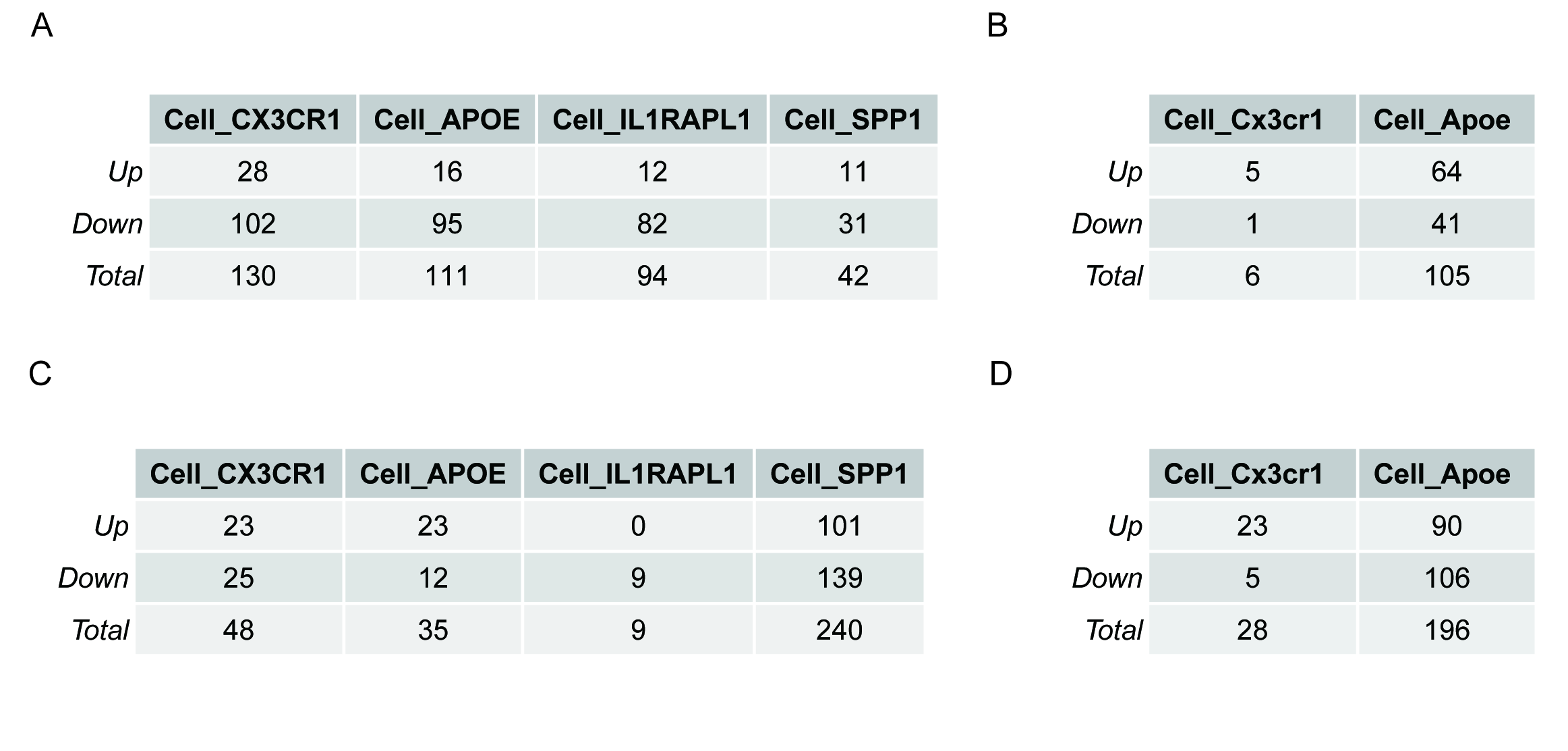


Figure S5 Comparisons of the numbers of up-regulated and down-regulated genes of DEGs between younger group and older group, or between NC group and AD group

1. The numbers of up-regulated and down-regulated genes of DEGs between younger group (age groups: 0.5_10, 11_20) and the older group (age group: 70_100) for subpopulations of microglia in humans (GSE198323)
2. The numbers of up-regulated and down-regulated genes of DEGs between younger group (age groups: 3, 6 months old) and the older group (age group: 21 months old) for subpopulations of microglia in mice (GSE127892)
3. The numbers of up-regulated and down-regulated genes of DEGs between NC group (age groups: 70_100) and the AD group (age group: 70_100) for subpopulations of microglia in humans (GSE198323)
4. The numbers of up-regulated and down-regulated genes of DEGs between APP_NL.F.G group (age groups: 12, 21 months old) and the C57Bl/6 group (age group: 12，21 months old) for subpopulations of microglia in mice (GSE127892)

### Figure S6


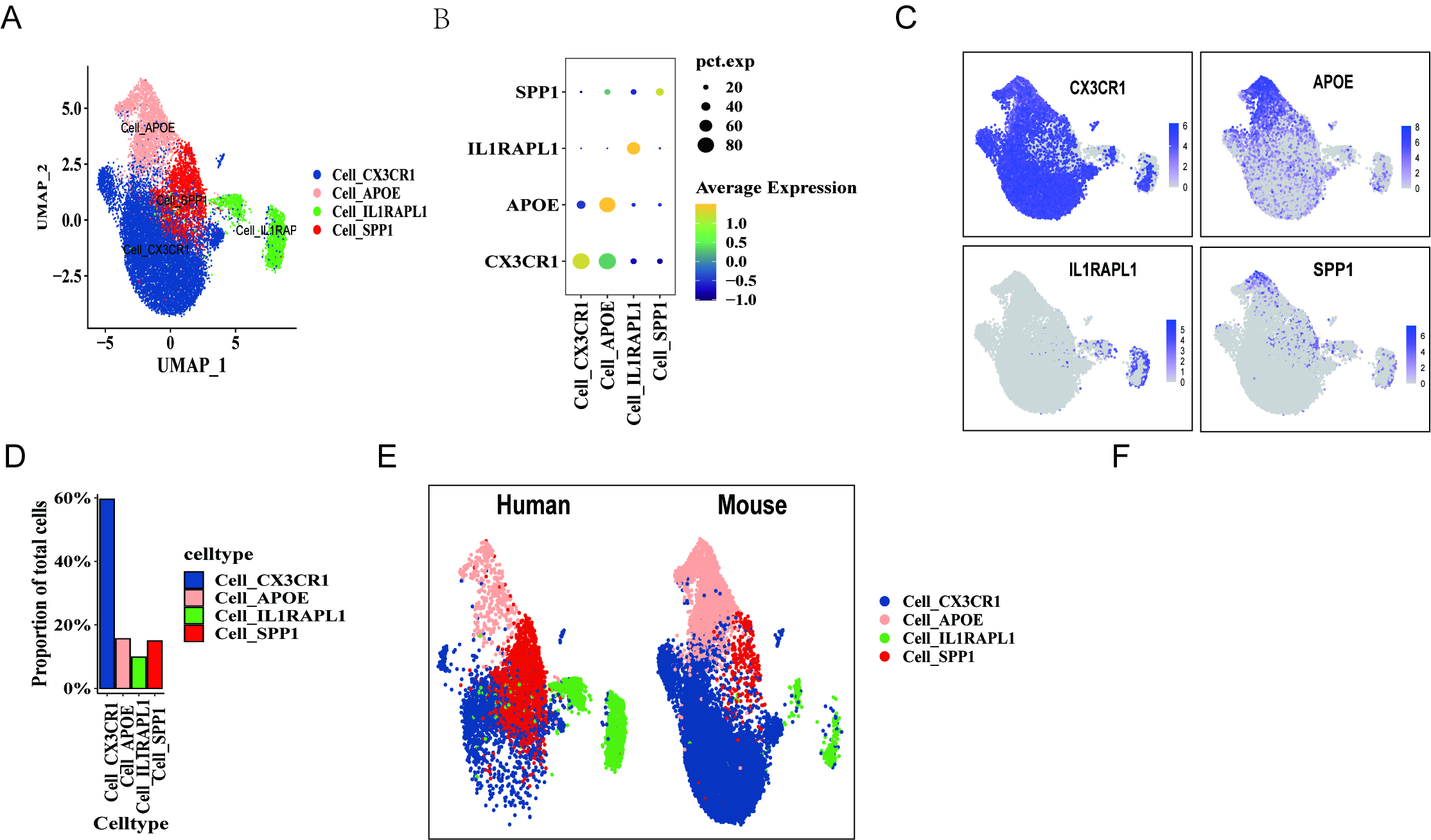


Figure S6 Cross-species analysis by using 14034 homologous genes of humans (GSE198323) and mice (GSE127892).

1. An UMAP plot exhibited distributions of 4 major subpopulations of microglia by using 14034 homologous genes of humans (GSE198323) and mice (GSE127892). 4 major subpopulations included Cell_ CX3CR1, Cell_APOE, Cell_IL1RAPL1 and Cell_SPP1.
2. A dotplot exhibited the expression for 4 marker genes (CX3CR1, APOE, IL1RAPL1 and SPP1) for 4 major subpopulations of microglia. CX3CR1, APOE, IL1RAPL1 and SPP1 as homologous genes of humans (GSE198323) and mice (GSE127892).
3. Distributions of 4 marker genes (CX3CR1, APOE, IL1RAPL1 and SPP1) for 4 major subpopulations of microglia. CX3CR1, APOE, IL1RAPL1 and SPP1 as homologous genes of humans (GSE198323) and mice (GSE127892).
4. The percentage of 4 major subpopulations of microglia by integrating humans (GSE198323) and mice (GSE127892).
5. An UMAP plot exhibited distributions of 4 major subpopulations of microglia split by species with using 14034 homologous genes of humans (GSE198323) and mice (GSE127892).

### Figure S7


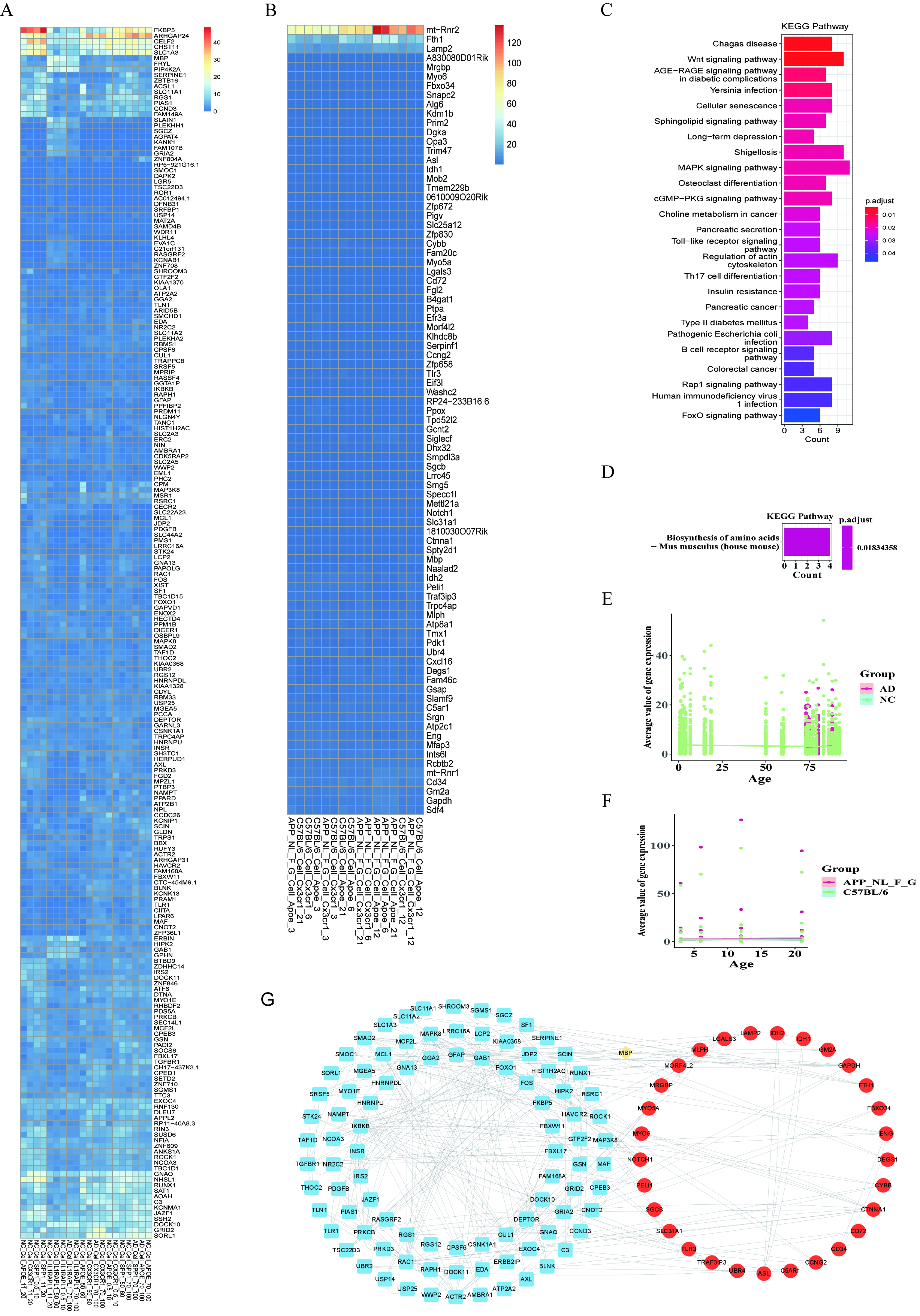


Figure S7 Comparison the genes from DEGs related to age, not to disease.

1. A heatmap exhibited the average expressions of 217 genes from DEGs which were age-related and not AD-related were obtained with p value <0.05 and |avg_log2FC|>0.5 for humans (GSE198323).
2. A heatmap exhibited the average expressions of 85 genes from DEGs which were age-related and not AD-related were obtained with p value <0.05 and |avg_log2FC|>0.5 for mice (GSE127892).
3. Pathways obtained from KEGG enrichment analyses by using 217 genes from DEGs which were age-related and not AD-related were obtained with p value <0.05 and |avg_log2FC|>0.5 for humans (GSE198323).
4. Pathways obtained from KEGG enrichment analyses by using 85 genes from DEGs which were age-related and not AD-related were obtained with p value <0.05 and |avg_log2FC|>0.5 for mice (GSE127892).
5. The average expressions of 217 genes changed with age by a scatterplot with linearly fitted curve. These 217 genes from DEGs which were age-related and not AD-related were obtained with p value <0.05 and |avg_log2FC|>0.5 for humans (GSE198323).
6. The average expressions of 85 genes changed with age by a scatterplot with linearly fitted curve. These 85 genes from DEGs which were age-related and not AD-related were obtained with p value <0.05 and |avg_log2FC|>0.5 for mice (GSE127892).
7. 92 DEGs in humans (blue rectangle shape), 29 genes in mice (red circle shape) and 1 gene shared in humans and mice (yellow diamond shape) exhibited close relationship between humans and mice by using method which was described in method section of supplementary materials.

### Figure S8


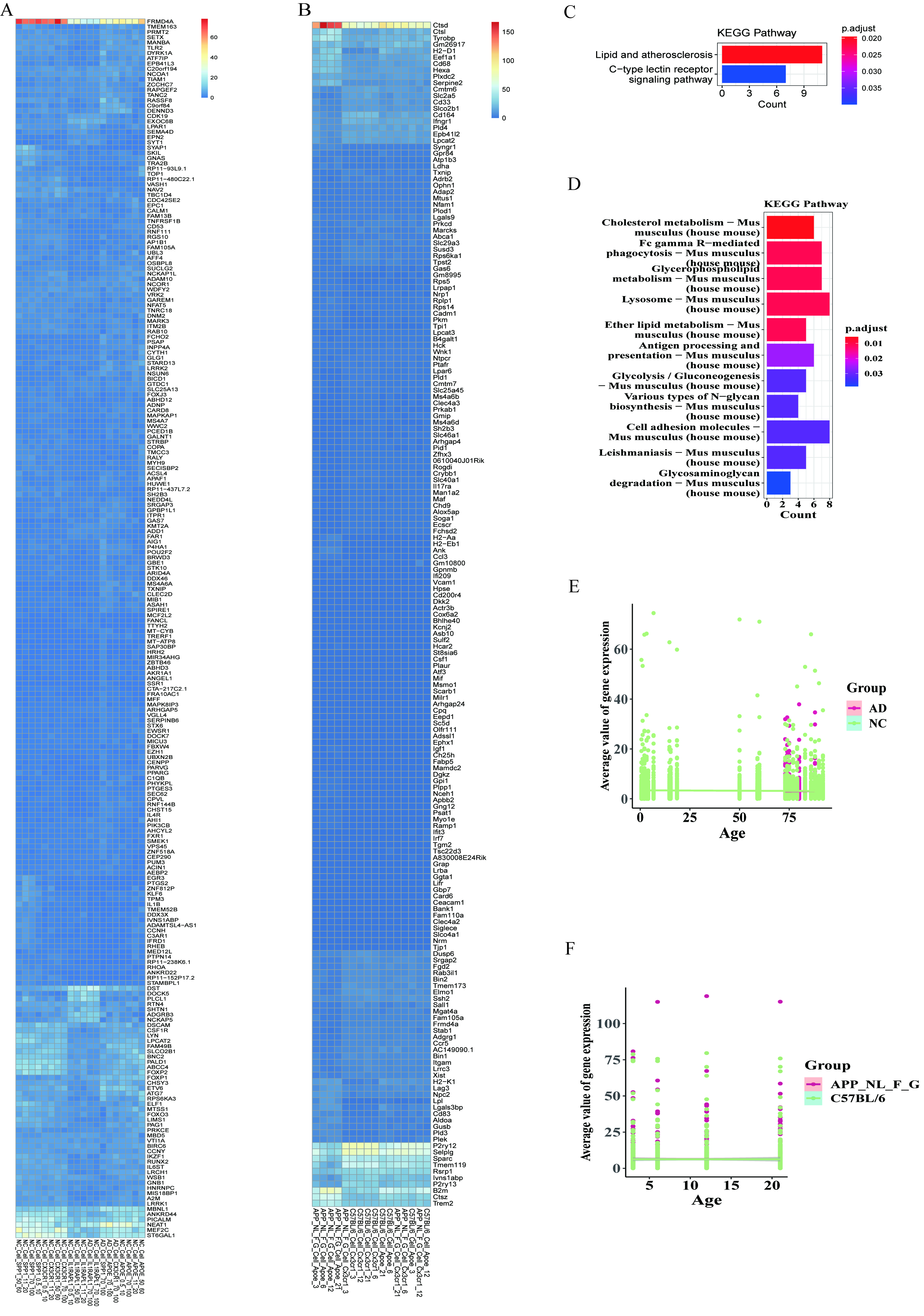


Figure S8 Comparison the genes from DEGs related to disease, not to age.

1. A heatmap exhibited the average expressions of 234 genes from DEGs which were AD-related and not age-related were obtained with p value <0.05 and |avg_log2FC|>0.5 for humans (GSE198323).
2. A heatmap exhibited the average expressions of 185 genes from DEGs which were AD-related and not age-related were obtained with p value <0.05 and |avg_log2FC|>0.5 for mice (GSE127892).
3. Pathways obtained from KEGG enrichment analyses by using 234 genes from DEGs which were AD-related and not age-related were obtained with p value <0.05 and |avg_log2FC|>0.5 for humans (GSE198323).
4. Pathways obtained from KEGG enrichment analyses by using 185 genes from DEGs which were AD-related and not age-related were obtained with p value <0.05 and |avg_log2FC|>0.5 for mice (GSE127892).
5. The average expressions of 234 genes changed with age by a scatterplot with linearly fitted curve. These 234 genes from DEGs which were AD-related and not age-related were obtained with p value <0.05 and |avg_log2FC|>0.5 for humans (GSE198323).
6. The average expressions of 185 genes changed with age by a scatterplot with linearly fitted curve. These 185 genes from DEGs which were AD-related and not age-related were obtained with p value <0.05 and |avg_log2FC|>0.5 for mice (GSE127892).

### Figure S9


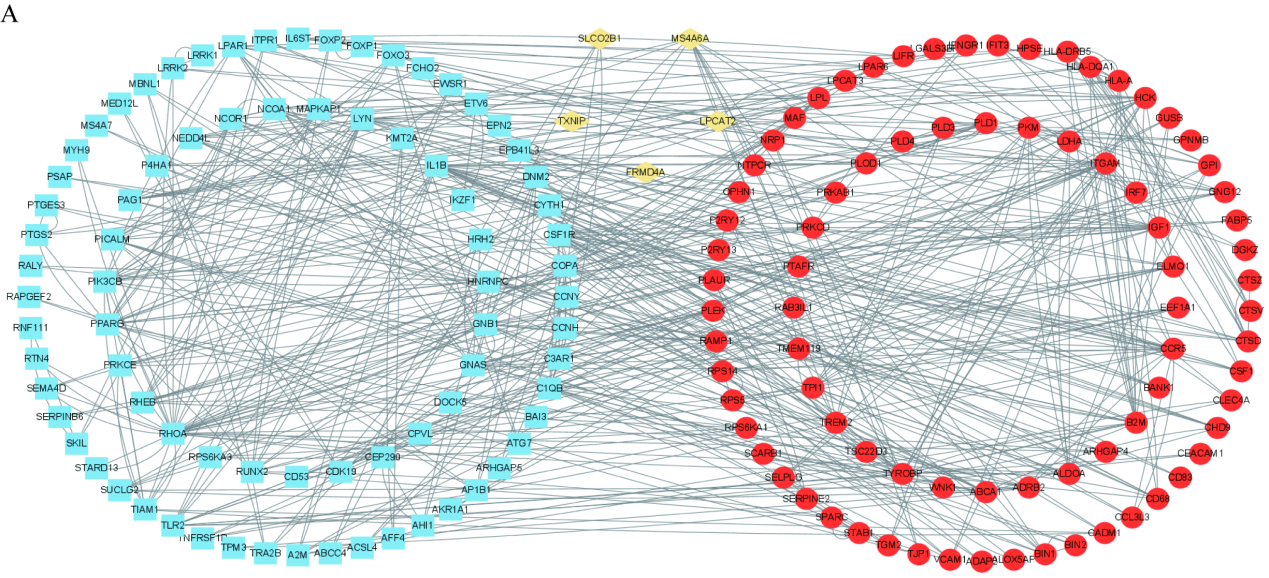


Figure S9 The association of genes from DEGs related to AD, not to age for humans and mices.

1. 79 genes in humans (blue rectangle shape), 81 genes in mice (red circle shape) and 5 genes (yellow diamond shape) shared in humans and mice exhibited close relationship between humans and mice by using method which was described in method section of supplementary materials

### Figure S10


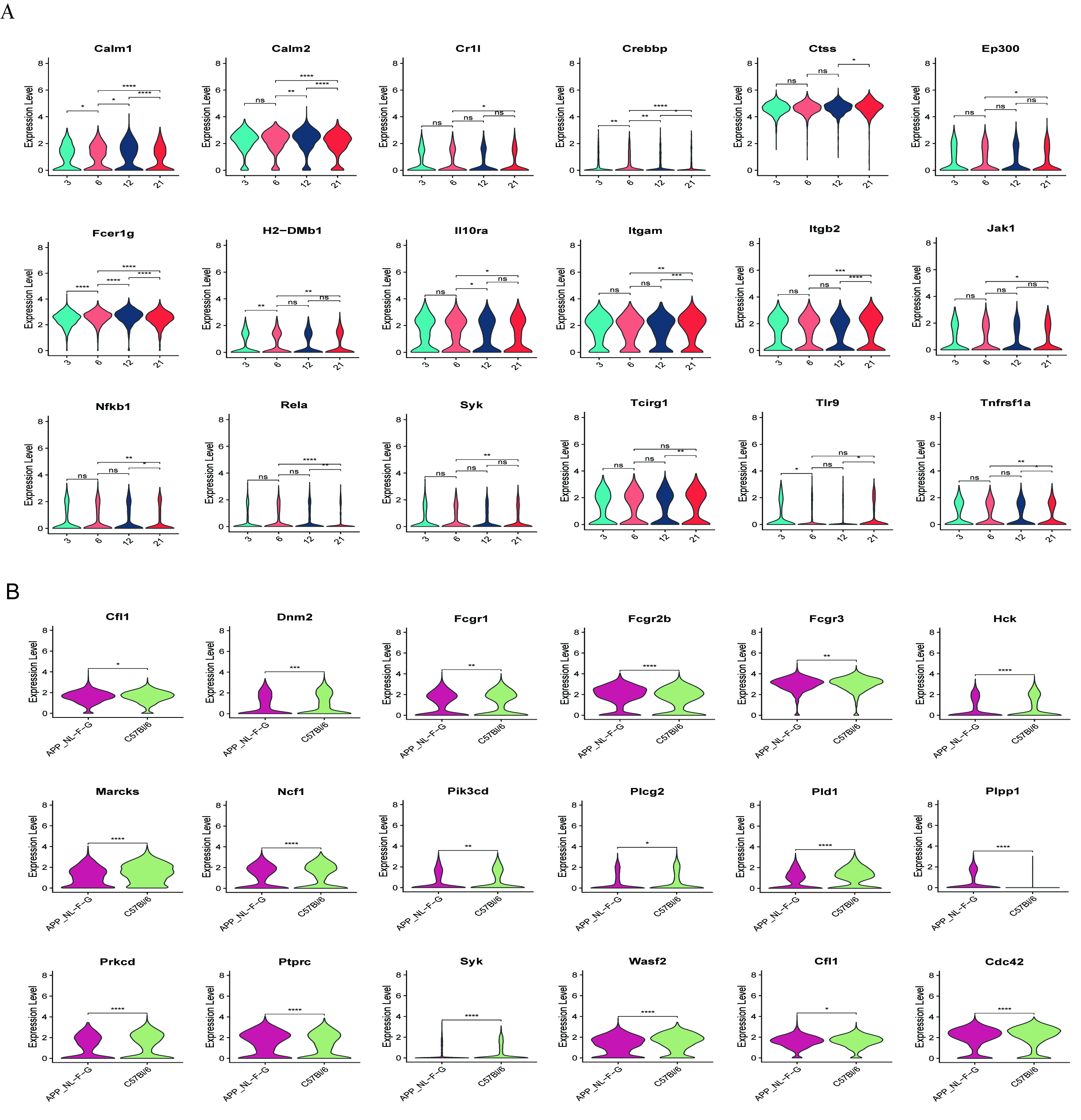


Figure S10 comparison of the expressions of genes in pathways shared in humans (GSE198323) and mice (GSE127892).

1. Comparison of the expressions of 12 genes in tuberculosis pathway according to age groups for mice(GSE127892). The tuberculosis pathway shared in microglia of humans (GSE198323) and mice (GSE127892) by using pseudotime analysis.
2. Comparison of the expressions of 18 genes in Fc gamma R−mediated phagocytosis pathway according to APP_NL-F-G and C57Bl/6 groups. The Fc gamma R−mediated phagocytosis pathway shared in microglia of humans (GSE198323) and mice (GSE127892) by using pseudotime analysis.
